# Genome-Wide Identification of SNF7 Gene Family in Maize and Potential Roles in Response to Abiotic and Biotic Stress

**DOI:** 10.3390/ijms27187985

**Published:** 2026-09-08

**Authors:** Dan Wang, Wei Hu, Cuiping Xin, Xinyan Sun, Wenbo Yang, Meichen Zhu, Huimin Li, Yanping Fan, Yanyong Cao

**Affiliations:** 1 State Key Laboratory of Maize Bio-Breeding, Institute of Cereal Crops, Henan Academy of Agricultural Sciences, Zhengzhou 450002, China; wangdan5567@126.com (D.W.); huwei2114@126.com (W.H.); xcp18838642579@163.com (C.X.); xinyansun123@163.com (X.S.); bbg.123@163.com (W.Y.); zmc910824@163.com (M.Z.); lihuimin19910218@126.com (H.L.); fyp5122009@163.com (Y.F.); 2 Henan Provincial Laboratory of Maize Biology, Engineering Research Center for Maize Genomic Editing and Genetic Transformation, Henan Academy of Agricultural Sciences, Zhengzhou 450002, China

**Keywords:** maize, *SNF7* gene family, ESCRT-III, gene expression, stress response

## Abstract

Sucrose non-fermenting protein 7 (SNF7) is a core operator of the endosomal sorting complex required for transport III (ESCRT-III) component mediating protein sorting and degradation. To date, the *SNF7* gene family remains poorly characterized in plants, particularly in maize (*Zea mays* L.). Here, we integrated bioinformatic and transcriptomic analyses to systematically characterize the *ZmSNF7* gene family and its regulatory potential in stress responses. In total, 20 *ZmSNF7* genes were identified genome-wide and classified into three phylogenetic clades, with conserved motifs and similar tertiary structures within the same clade. Abundant hormone- and stress-responsive cis-elements were detected in their promoters. Protein interaction prediction indicated ZmSNF7 proteins interact with intra-family members and other ESCRT components. Gene Ontology (GO) enrichment analysis suggested ZmSNF7s are primarily involved in endomembrane system organization and vesicular trafficking. Transcriptomic data revealed divergent *ZmSNF7* expression patterns under drought, Rice black-streaked dwarf virus (RBSDV) infection, *Colletotrichum graminicola* (*C. graminicola*) inoculation and Asian corn borer (ACB) infestation. Collectively, this study comprehensively characterized the *ZmSNF7* gene family and broadened our functional understanding of *ZmSNF7* in mediating plant responses to biotic and abiotic stresses.

## 1. Introduction

Maize is one of the most widely cultivated food crops globally, and also serves as a crucial raw material for animal feed and industrial production. Maize is highly susceptible to a broad spectrum of abiotic stresses (e.g., drought, high temperature) and biotic stresses (e.g., viruses, bacteria, fungi, and insect pests) throughout its growth cycle, which cause severe yield losses and quality deterioration. In China, seasonal outbreaks of multiple maize diseases and pests—including maize rough dwarf disease [1], southern corn rust [2], stalk rot [3], ear rot [4], corn anthracnose [5], Asian corn borer (*Ostrinia furnacalis*) [6], oriental armyworm, and fall armyworm [7]—occur annually. These recurrent outbreaks not only severely constrain the improvement of maize yield and quality but also impose substantial economic losses on agricultural producers. Accordingly, the identification and functional characterization of stress resistance genes have become a core research priority in maize breeding.

The endosomal sorting complex required for transport (ESCRT) is a structurally and functionally conserved complex in eukaryotic cells. It regulates the formation of intraluminal vesicles (ILVs) inside multivesicular bodies/prevacuolar compartments (MVBs/PVCs) [8]. Its primary biological function is to promote the degradation of ubiquitin-tagged membrane proteins [9]. Furthermore, ESCRT is involved in diverse physiological processes, including cell division [8], viral budding [10], autophagy [11,12] and immune defense responses [13]. Recent studies have also demonstrated that ESCRT participates in plasma membrane (PM) repair [14] and nuclear envelope restoration [15].

In plants, the *SNF7* gene family, a core subunit of the ESCRT complex, exhibits prominent functional pleiotropy and a sophisticated regulatory network, participating in a wide range of critical biological processes such as leaf senescence [16], sorting and degradation of ubiquitinated proteins [17], stomatal patterning [18], and autophagic turnover of chloroplast components [19]. Specifically, rice *OsSNF7.2* (Os09g0267600) modulates leaf rolling morphogenesis by mediating the endosomal trafficking and turnover of the auxin biosynthetic enzyme OsYUC8, thereby directly shaping key agronomic traits including plant architecture and photosynthetic efficiency [20]. In *Arabidopsis thaliana*, induced expression of a dominant-negative ESCRT-III mutant triggers severe cotyledon developmental defects, coupled with reduced mesophyll cell volume, impaired central vacuole biogenesis, and aberrant chloroplast development [21]. By binding to other ESCRT-III subunits, SNF7 anchors itself to multivesicular bodies and mediates the vacuolar trafficking of the phosphate transporter PHT1;1 in *Arabidopsis thaliana*. *SNF7* partial loss-of-function mutants exhibit a remarkable reduction in the vacuolar degradation level of PHT1;1 [22]. Collectively, these findings confirm that the ESCRT complex serves as a critical regulator of endosomal sorting and ubiquitinated protein turnover in plants, and exerts pivotal functions in plant morphogenesis, organellar homeostasis maintenance, and the modulation of key agronomic traits.

SNF7 participates in the regulation of intracellular protein degradation and thus plays a vital role in plant immune responses. Knockout of the *SNF7* gene reduces the RNA replication level of Brome mosaic virus by more than 25-fold and completely inhibits the formation of detectable viral spherules. This inhibitory effect is observed even when viral replication-associated proteins accumulate normally and are targeted to the perinuclear endoplasmic reticulum membrane [23]. Additionally, *NbSNF7* is involved in the infection process of Tomato spotted wilt virus in plants [24].

The complete ESCRT system comprises five core protein complexes: ESCRT-0, ESCRT-I, ESCRT-II, ESCRT-III and the vacuolar protein sorting 4 (VPS4)–VPS twenty-associated 1 (VTA1) complex. The molecular mechanism by which ESCRT drives membrane invagination is generally summarized into three steps. Firstly, ESCRT-I and ESCRT-II bind to the endosomal membrane and trigger membrane invagination to form initial buds. Secondly, ESCRT-III accumulates at the bud neck and mediates bud scission, which releases intraluminal vesicles into the endosomal cavity and ultimately gives rise to mature MVBs. Lastly, the VPS4-VTA1 complex hydrolyzes adenosine triphosphate (ATP) to provide energy for disassembling the assembled ESCRT-III complex, thereby enabling its cyclic reuse to support continuous membrane budding [25]. In plant cells, the large ESCRT-III membrane scaffold complex is assembled from SNF7, VPS2 and the accessory proteins VPS46 and VPS60, the majority of which are encoded by members of the *SNF7* gene family [26].

As core components of ESCRT-III, SNF7 family proteins are primarily responsible for vesicle scission [27]. The *SNF7* gene family was first identified in yeast. Although *SNF7* genes have been extensively characterized in yeast and mammals, relevant research in plants remains limited, and the *SNF7* gene family has not yet been systematically investigated in plant species [28]. In this study, genome-wide identification of *ZmSNF7* genes was performed using the maize inbred line B73. Multiple bioinformatics analyses were further conducted, including chromosomal localization, gene structure characterization, conserved motif prediction, cis-acting element analysis, GO enrichment analysis, and protein interaction network prediction. By integrating transcriptome data, we explored the expression profiles of *ZmSNF7* genes across diverse tissues and organs, as well as under abiotic and biotic stress conditions. This study aims to clarify the evolutionary features and functional characteristics of *ZmSNF7* genes. The findings provide a theoretical foundation for revealing the regulatory roles of *ZmSNF7* in response to biotic and abiotic stresses, and offer reference data for functional research of *ZmSNF7* in maize.

## 2. Results

### 2.1. Identification and Characterization of ZmSNF7 Genes in Maize

In this study, a total of 20 *SNF7* family genes in maize, 15 in rice and 14 in Arabidopsis were identified through genome-wide BLASTP and HMMER searches. Pfam analysis verified that all these genes encode proteins harboring the canonical SNF7 domain (PF03357). Significant variations were observed in the amino acid length (100–411 residues) and molecular weight (11.58–46.56 kDa) among ZmSNF7 protein members (Table 1 and Table S1). The pI (theoretical isoelectric point) values of ZmSNF7 proteins exhibited a multimodal distribution with prominent peaks at strongly acidic and strongly basic ranges. Notably, the ZmSNF7 family proteins generally displayed high instability indices and negative GRAVY (grand average of hydropathicity) values. This combination of charge diversity, high instability, and strong hydrophilicity enables SNF7 proteins to not only adapt to the charge regulatory demands of distinct subcellular microenvironments but also support the assembly/disassembly of the ESCRT-III complex through their dynamic turnover properties, thereby mediating multiple membrane remodeling functions.

Signal peptides and subcellular localization predictions revealed that none of these maize SNF7 proteins contain a secretory signal peptide, a mitochondrial/chloroplast transit peptide, or a thylakoid transit peptide (Table S2). Additionally, no N-terminal leader peptide or corresponding cleavage site was detected (Table S3). Collectively, no typical organelle-targeting signal peptides were predicted for maize SNF7 proteins.

### 2.2. Phylogenetic Analysis and Classification of the SNF7 Gene Family

**Figure 1:**
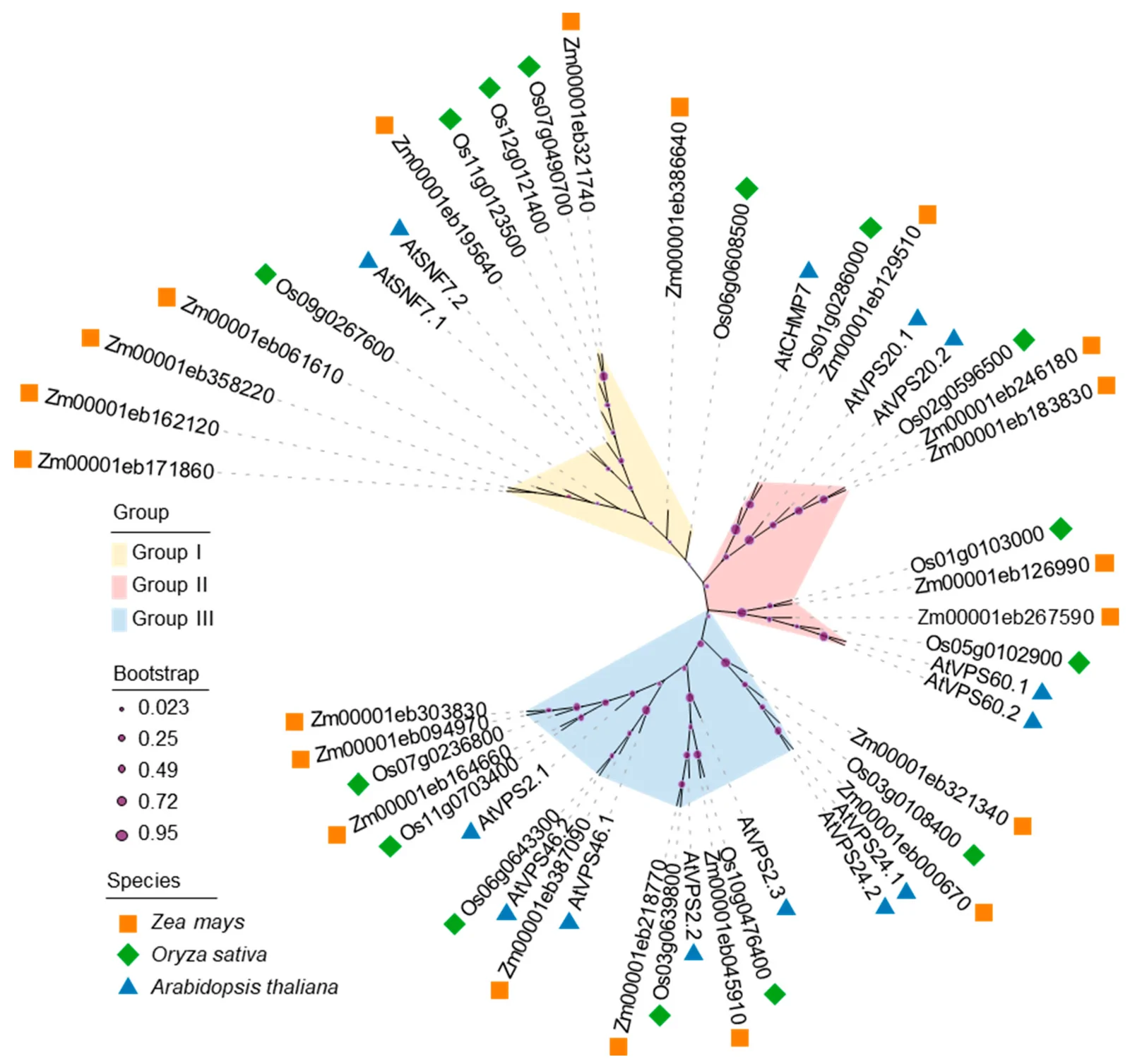
Phylogenetic analysis of SNF7 proteins in maize, rice and Arabidopsis. Three groups are marked with different colors. ZmSNF7, OsSNF7, and AtSNF7 proteins are represented by orange squares, green diamonds, and blue triangles, respectively.

We performed multiple sequence alignment and constructed a phylogenetic tree (Figure 1) using the amino acid sequences of SNF7 family proteins of maize, rice, and Arabidopsis. Phylogenetic analysis classified the ZmSNF7, OsSNF7 and AtSNF7 proteins into three distinct subgroups (Group I–III). Group I, Group II and Group III comprised 7, 5 and 8 ZmSNF7 members, 5, 4 and 6 OsSNF7 members, and 2, 5 and 7 AtSNF7 members, respectively. Each subgroup harbored members from all three species, indicating that the divergence of these SNF7 clades predated species differentiation, supporting the evolutionary conservation of this gene family. *ZmSNF7* genes were the most numerous and distributed across all three subgroups, whereas *OsSNF7* and *AtSNF7* homologs were dispersed in different clades.

### 2.3. Gene Structure and Conserved Motif Analysis of ZmSNF7 Genes

**Figure 2:**
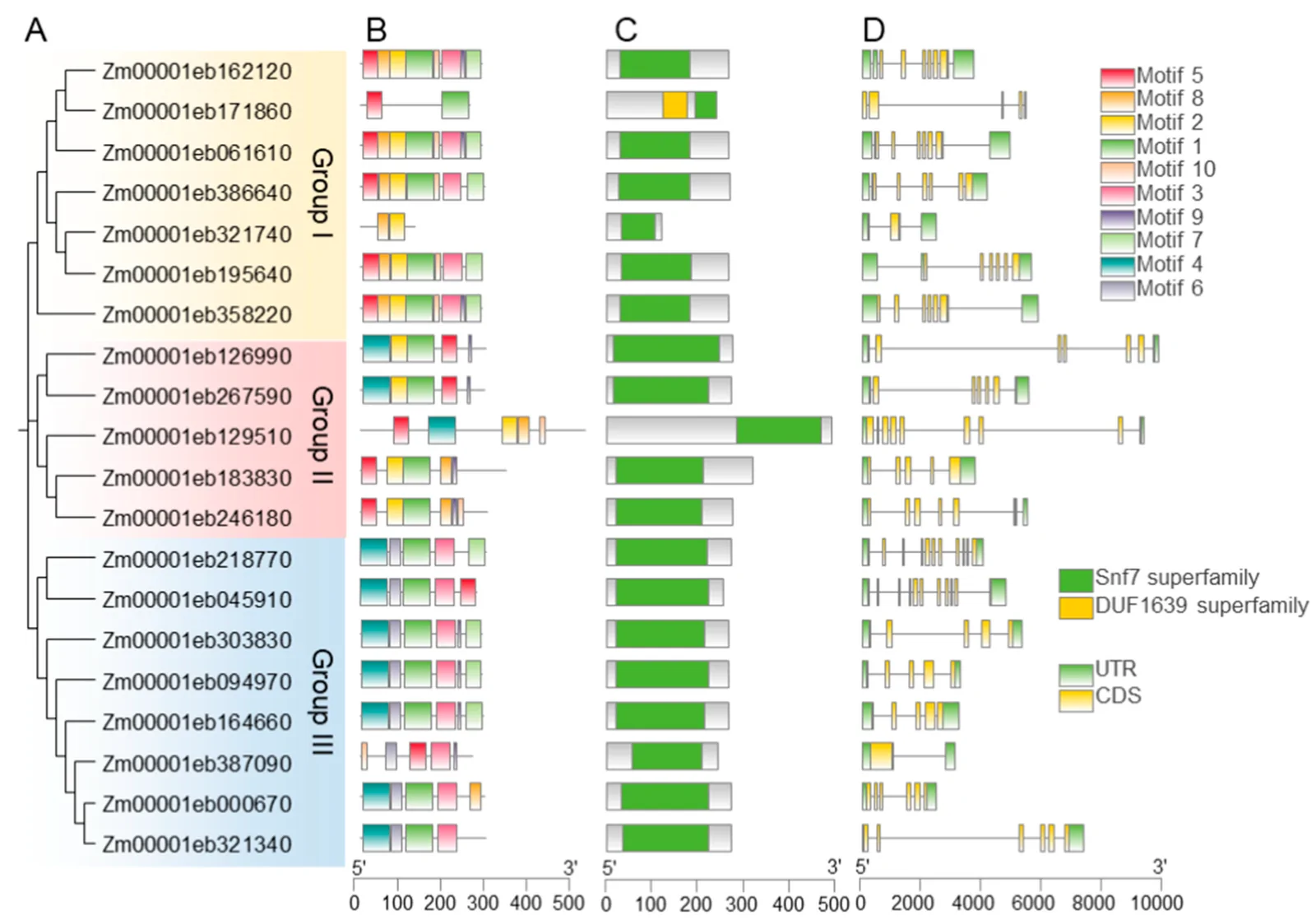
Conserved motifs, conserved domains, and gene structures of *ZmSNF7s*. (**A**) Phylogenetic tree. (**B**) Distribution of conserved motifs. (**C**) Conserved domains composition. (**D**) Gene structures of *ZmSNF7* genes.

Phylogenetic analysis classified the 20 *ZmSNF7* family members into three subgroups (Figure 2A). Conserved motif analysis (Figure 2B) revealed that Motifs 1, 3, 5, and 7 were shared by most members, constituting the core conserved sequences. Group II and III harbored the specific motif Motif 4, and some members of Group III additionally contained Motifs 4 and 6, with variations in motif composition being highly consistent with the phylogenetic grouping. The sequence logos illustrating amino-acid conservation for each motif are shown in Figure S1. Prediction of conserved motifs showed that motif composition and arrangement of SNF7 family proteins from maize, rice and *Arabidopsis thaliana* are highly conserved between monocots and dicots, reflecting structural and functional stability of this family during evolution (Figure S2).

Conserved domain analysis (Figure 2C) showed that all ZmSNF7 proteins contained the Snf7 superfamily domain (PF03357), while only *Zm00001eb171860* in Group I carried an additional DUF1639 domain, suggesting potential functional divergence of this member. Gene structure analysis (Figure 2D) indicated that the intron length, exon-intron organization, and UTR/CDS distribution of *ZmSNF7* family members were highly heterogeneous, with no obvious commonalities among subgroups.

The predicted three-dimensional structures (Figure S3) of the 20 ZmSNF7 proteins exhibited a highly conserved core α-helical fold across all three phylogenetic subgroups, with apparent structural variations mainly originating from differences in the flexible N- and C-terminal intrinsically disordered regions rather than the functionally constrained ESCRT-III core domain.

### 2.4. Chromosomal Distribution, Synteny Analysis and Evolutionary Dynamics

**Figure 3:**
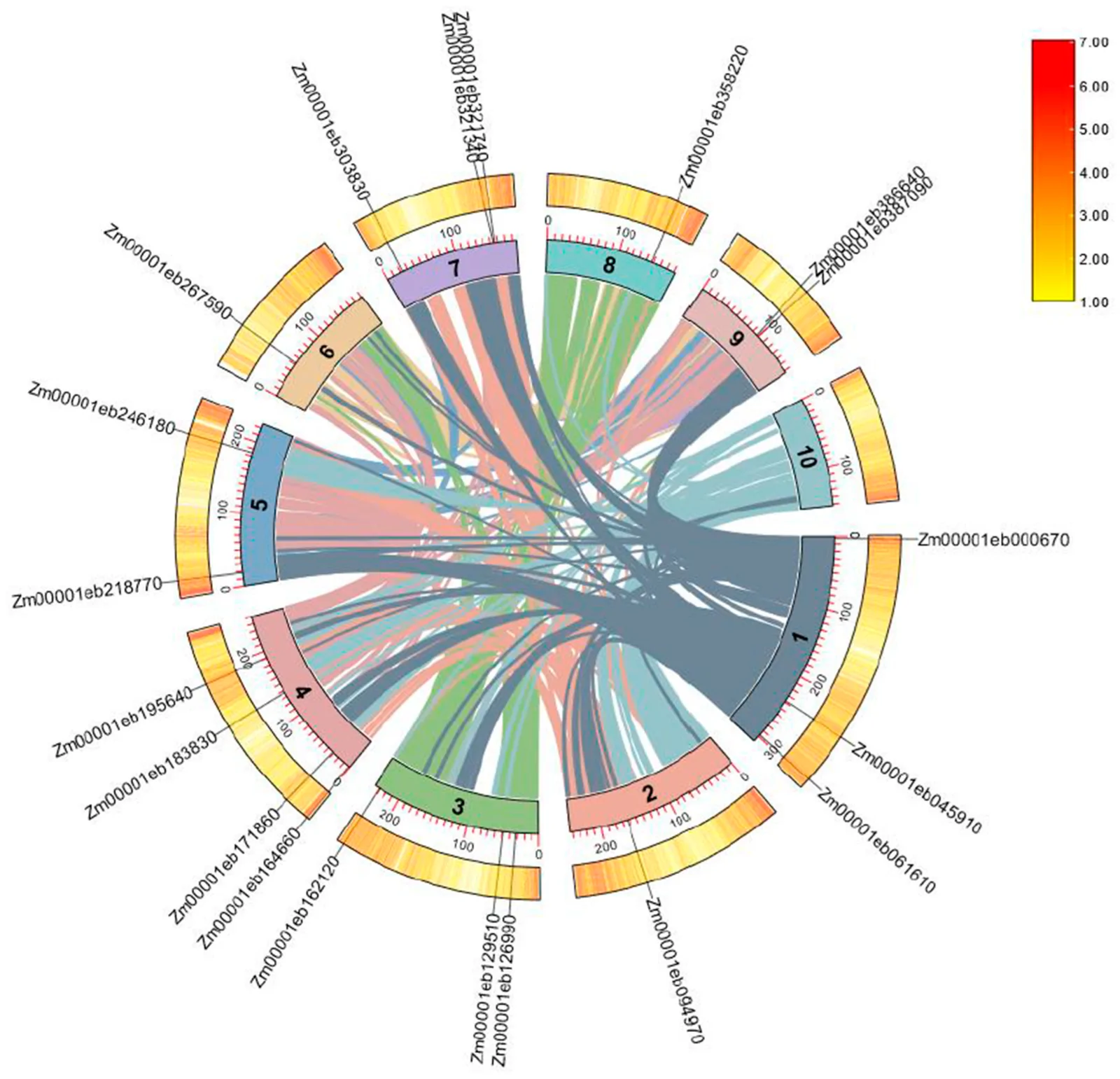
Synteny relationship, chromosome density and chromosomal locations of *ZmSNF7s* genes in maize. Note: numbers 1–10 indicate 10 chromosomes. Scale bar on the circle indicates the chromosome length (Mb).

The 20 maize *ZmSNF7* family genes were unevenly distributed across nine of the ten maize chromosomes. Some chromosomes harbor multiple members, whereas others carry only 1–2 genes, and chromosome 10 bears no *ZmSNF7* family members (Figure 3).

We analyzed tandem/segmental duplications and Ka/Ks ratios of maize *ZmSNF7* paralogs to investigate family expansion and selection pressures. No tandem or segmental duplications were detected, indicating a conserved evolutionary pattern with no expansion via these canonical routes. All paralog pairs showed Ka/Ks < 1, reflecting strong purifying selection with no positive selection signals. These findings align with SNF7′s core ESCRT complex function: sequences and copy numbers are under strict functional constraints, critical for processes like endocytosis and membrane repair.

Genome-wide collinearity analysis of the *ZmSNF7* gene family across B73, Mo17, and teosinte SK was conducted to characterize its homology distribution (Figure S4). The vast majority of the 20 family members (19/20, 95%) exhibited collinear orthologous regions in all three genomes, reflecting high evolutionary conservation across maize subspecies and inbred lines. Notably, only one member (*Zm00001eb171860*) lacked collinear orthologs in both Mo17 and SK.

### 2.5. Cis-Acting Element Analysis of ZmSNF7 Gene Promoters

**Figure 4:**
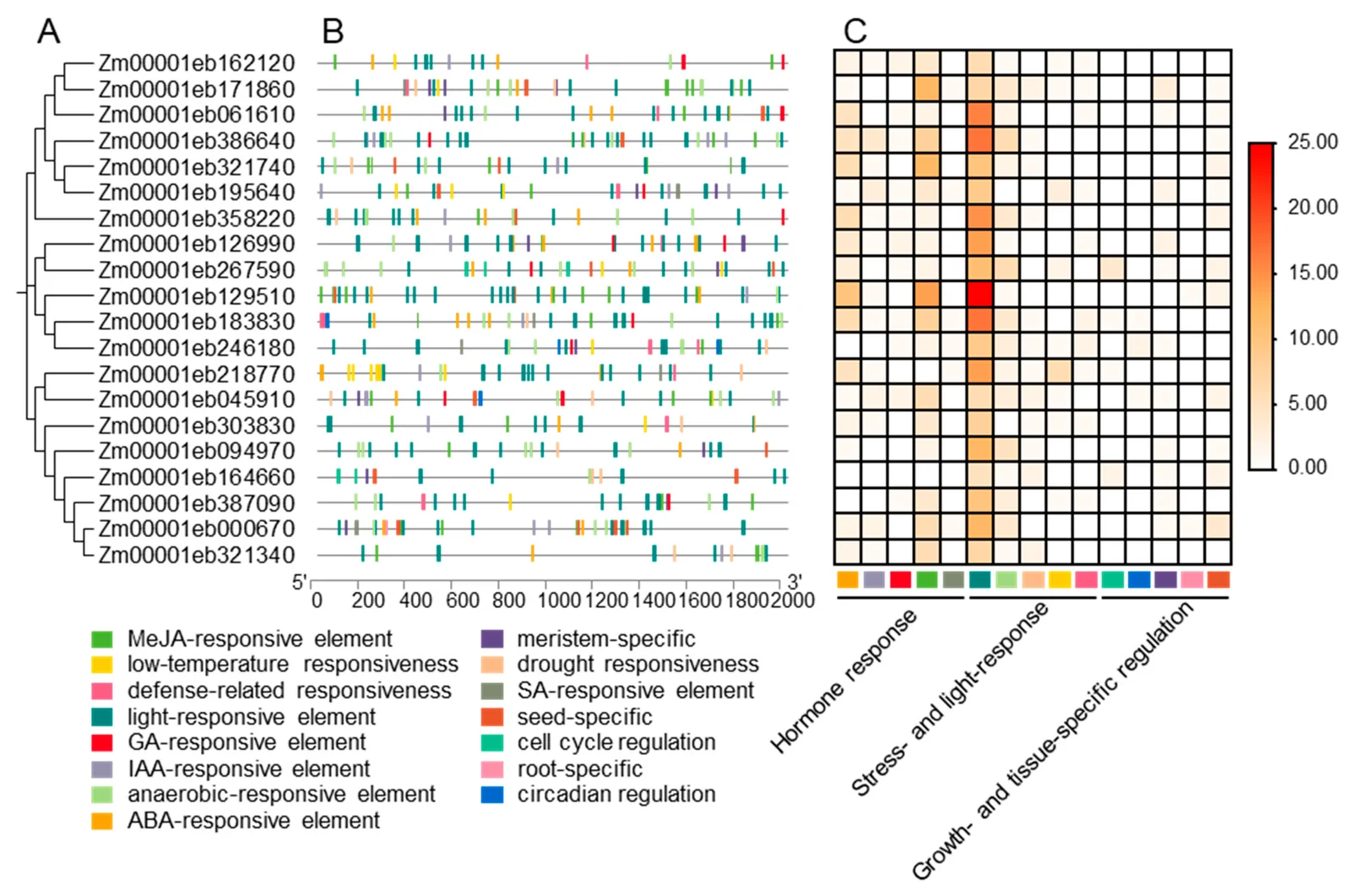
Analysis of cis-acting elements of the *ZmSNF7s* gene family. (**A**) Phylogenetic tree. (**B**) Distribution pattern of cis-acting elements. (**C**) Heatmap of the quantity statistics of cis-acting elements.

To investigate the potential regulatory mechanisms of *ZmSNF7* family genes, we analyzed their cis-acting elements (Figure 4A–C). Phylogenetic analysis revealed the evolutionary relationships among the 20 identified *ZmSNF7* members. Promoter element analysis uncovered diverse motifs, including phytohormone-responsive (ABA-, MeJA-, SA-, GA-, IAA-responsive), stress-responsive (drought, low-temperature, light, anaerobic), tissue-specific (root-, seed-, meristem-specific), and development-related (cell cycle, circadian regulation) elements. Notably, both ABA-responsive and MeJA-responsive elements were widely distributed across most *ZmSNF7* promoters.

### 2.6. Protein Interaction Network and Functional Annotation Analysis of ZmSNF7s

**Figure 5:**
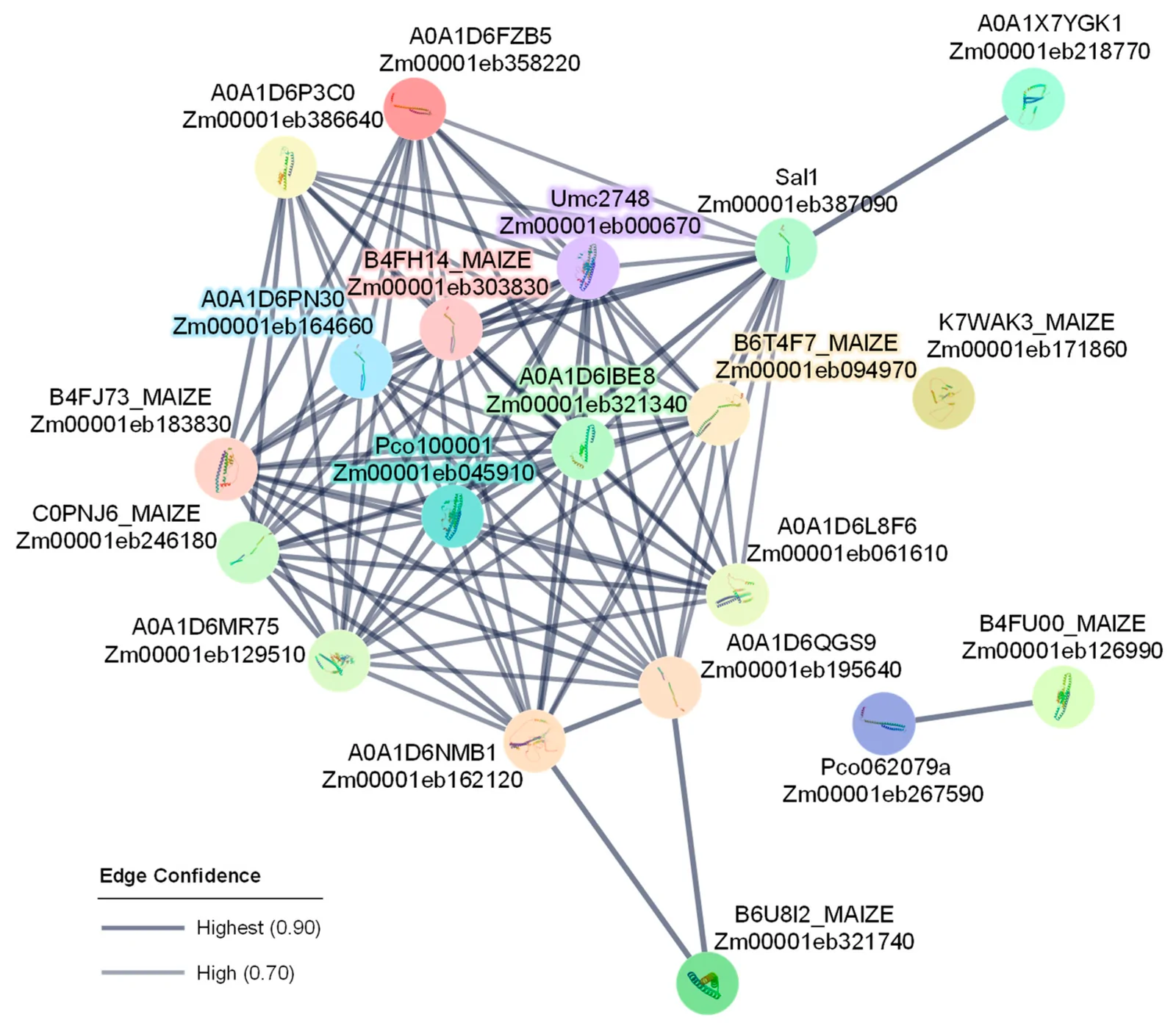
Analysis of the protein interaction network of ZmSNF7s. All data were obtained from the STRING database.

Using the STRING database, with interaction evidence derived from experimental validations and curated database entries, we analyzed the predicted protein–protein interaction (PPI) network of 20 ZmSNF7 family proteins (Figure 5). Our results revealed extensive interconnections among most ZmSNF7 members. Proteins such as A0A1D6FZB5 and A0A1D6P3C0 occupied central hub positions in the network, interacting with numerous other family members. In contrast, proteins including A0A1 × 7YGK1 and B4FU00_MAIZE showed only limited interactions or appeared as isolated nodes. Notably, K7WAK3_MAIZE (*Zm00001eb171860*) exhibited no predicted interactions with any other ZmSNF7 protein in the network.

PPI prediction for the target ZmSNF7 protein (B4FU00_MAIZE) was conducted using the STRING database (Figure S5). The results revealed that ZmSNF7 predominantly interacts with canonical ESCRT pathway components, including VPS24, VPS32, charged multivesicular body protein 1B (CHMP1B), CHMP5 and CHMP4b. Two AAA ATPase family members, suppressor of K^+^ transport growth defect 1 (SKD1) and vacuolar sorting protein 4b (VPS4b), were identified as interacting partners, which mediate ESCRT-III complex disassembly and cyclic reuse. Multiple LYST-interacting protein 5-like proteins, the positive regulators of VPS4/SKD1 activity, were also enriched in the interaction network (Table S4).

Cross-species sequence conservation analysis of 20 ZmSNF7 proteins using the STRING database revealed that these proteins exhibit high sequence conservation predominantly in eukaryotes, with negligible conservation in bacteria and archaea. These findings suggest that the *ZmSNF7* family is functionally conserved throughout eukaryotic evolution (Figure S6). Within the green plant clade, all *ZmSNF7* family proteins correspond to intense red blocks on the heatmap. This observation indicates that this gene family has been broadly retained during plant evolution, and its functions are essential for plant survival.

GO enrichment analysis (Figure S7) revealed that *ZmSNF7* family genes were markedly enriched in biological processes associated with intracellular vesicle trafficking, including vacuolar transport, endosomal sorting, vesicle-mediated transport and endomembrane system organization. For cellular component terms, these genes were predominantly annotated to vacuole, vesicle, membrane-bound organelle and membrane protein complex.

### 2.7. Spatial Expression Analysis of ZmSNF7s

**Figure 6:**
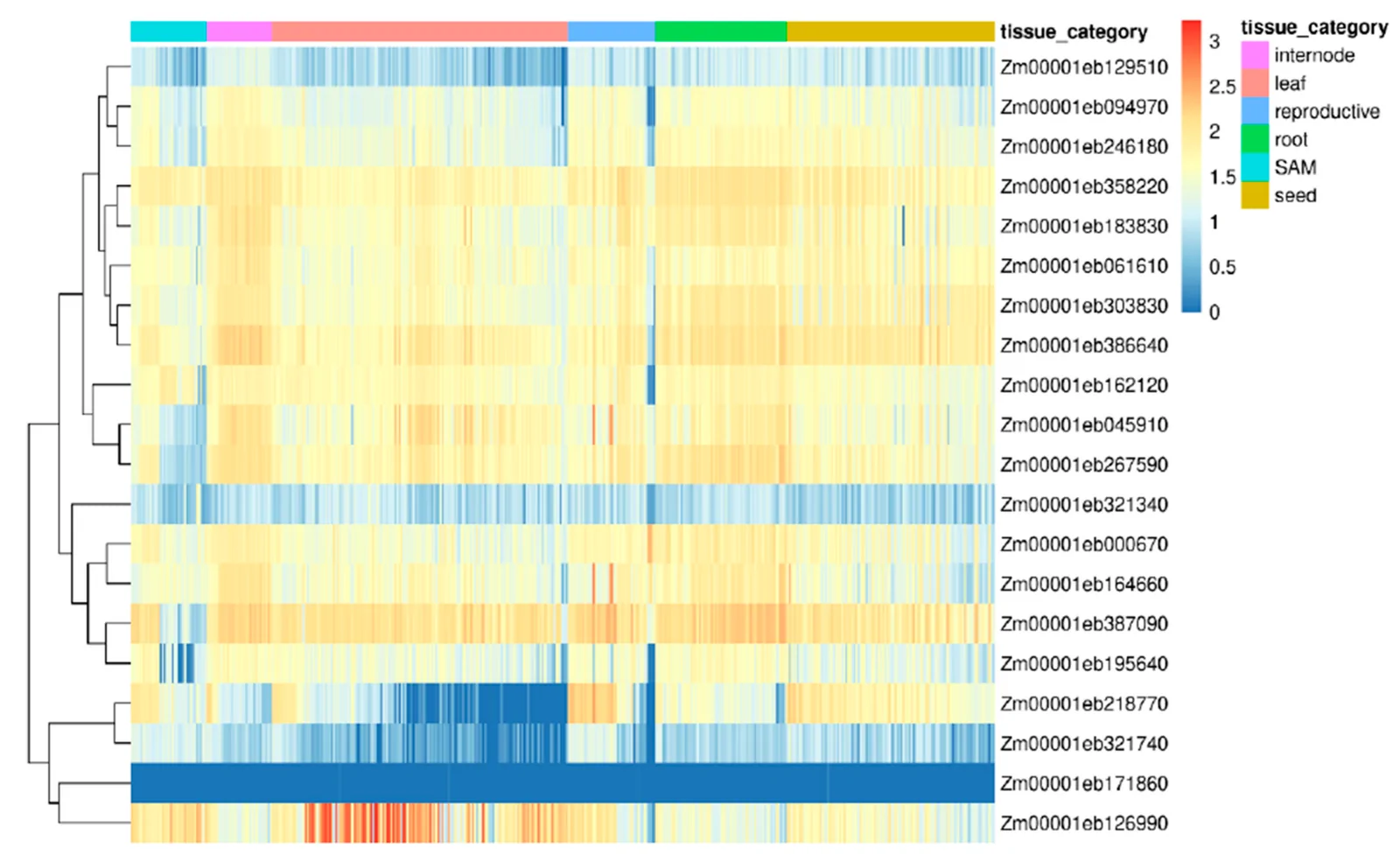
Expression heatmap of *ZmSNF7* family genes in different maize tissues. Different colors represent different tissue categories, including internode, leaf, reproductive tissues, root, shoot apical meristem (SAM), and seed.

To dissect the tissue expression profiles of the *ZmSNF7* gene family in maize, we generated a transcriptome-based expression heatmap. The majority of *ZmSNF7* members displayed broad, moderate expression across all assayed tissues, whereas *Zm00001eb171860* exhibited nearly undetectable transcript abundance in all examined tissues. A small subset of genes showed pronounced tissue-specific high expression; specifically, *Zm00001eb126990* was predominantly expressed in leaf tissues (Figure 6).

### 2.8. Expression Analysis of ZmSNF7s Under Abiotic and Biotic Stresses

**Figure 7:**
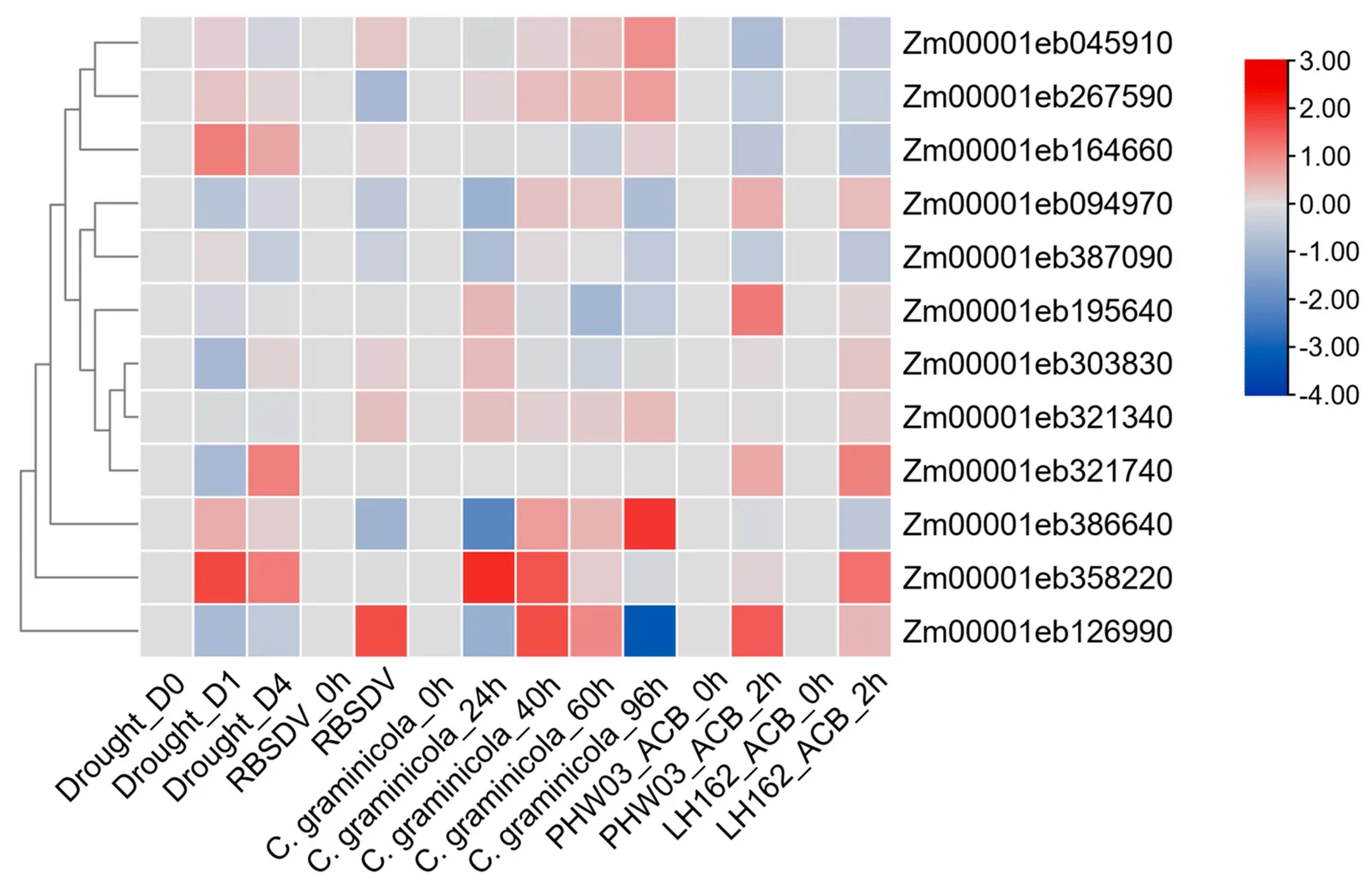
Expression heatmap of *ZmSNF7* family genes in different stress conditions. Genes with negligible expression in control samples were excluded from analysis. Label abbreviations: Drought_D0/D1/D4 = drought treatment at Day 0 (control), Day 1 and Day 4; RBSDV_0h = virus-free control at 0 h; *C. graminicola* = *Colletotrichum graminicola*, samples collected at 0, 24, 40, 60, 96 h post-inoculation; PHW03 and LH162 are two maize lines; PHW03_ACB_0h = undamaged control, PHW03_ACB_2h and LH162_ACB_2h = samples harvested at 2 h post-ACB infestation. h, hours.

Plants encounter a wide spectrum of biotic and abiotic stresses during growth and development. To elucidate the potential biological functions of *ZmSNF7* genes under stress conditions, we analyzed their expression response profiles to drought, viral infection, fungal pathogen challenge, and insect herbivory using four publicly available transcriptome datasets. After filtering out genes with extremely low expression levels in the control group, the remaining 12 *ZmSNF7* paralogous genes were retained for subsequent analyses. The results showed that *ZmSNF7* genes exhibited diverse expression patterns across different stress conditions (Figure 7, Table S5).

Following 1 day of drought stress treatment in B73 seedlings, *Zm00001eb164660* and *Zm00001eb358220* were significantly upregulated by over 2-fold at 1 day post-treatment, whereas their induction magnitudes declined by Day 4. This expression pattern was corroborated by quantitative real-time PCR (qPCR) validation (Figure S8A).

When challenged with RBSDV, *Zm00001eb126990* was significantly upregulated by over 3-fold, whereas *Zm00001eb386640* exhibited a more than 2-fold reduction in transcript abundance.

Upon inoculation with *C. graminicola*, individual *ZmSNF7* genes exhibited distinct transcriptional regulatory profiles. *Zm00001eb358220* was strongly induced by more than 4-fold at 24 h post-inoculation (hpi); its expression level then gradually decreased and nearly returned to the pre-inoculation baseline at 96 hpi. In comparison, the transcript abundance of *Zm00001eb267590* increased continuously starting from 24 hpi. In qPCR assays, no significant differential expression of *Zm00001eb358220* was detected in maize seedlings after *Fusarium verticillioides* inoculation, while *Zm00001eb267590* was significantly upregulated at 2 days post-inoculation (Figure S8B).

Two maize genotypes, PHW03 (highly susceptible to Asian corn borer, ACB) and LH162 (highly resistant to ACB), showed consistent transcriptional responses (both upregulation and downregulation) of *ZmSNF7* genes at 2 h post-ACB infestation. For example, *Zm00001eb126990* and *Zm00001eb195640* were significantly induced by 2- to 3-fold, whereas *Zm00001eb045910* and *Zm00001eb164660* were substantially downregulated. The qPCR experiments were performed using the maize inbred line B73, which is genotypically distinct from the two inbred lines used for transcriptome analysis, resulting in divergent findings. Specifically, *Zm00001eb126990* was significantly upregulated and *Zm00001eb045910* was significantly downregulated after ACB infestation, while *Zm00001eb164660* and *Zm00001eb195640* showed no significant expression changes in response to ACB treatment (Figure S8C).

Furthermore, FPKM quantification was performed on transcriptome data of the four stress treatments, and bar/line charts were generated to characterize transcriptional alterations of *ZmSNF7* genes from an alternative perspective. Under drought stress, the expression patterns of these genes were roughly classified into four categories, including initial downregulation followed by upregulation, early upregulation followed by repression, and sustained upregulation (Figure S9). Upon RBSDV infection, ten *ZmSNF7* paralogs were significantly upregulated (*p* < 0.05; Figure S10). Distinct temporal transcriptional responses to *C. graminicola* were observed among *ZmSNF7* genes (Figure S11). Some genes were continuously upregulated and maximally expressed at 96 h, while others displayed transient induction or fluctuating expression profiles over time. After infestation of maize inbred line PHW03 with ACB, three *ZmSNF7* genes were markedly downregulated, whereas six were significantly induced (Figure S12).

## 3. Discussion

### 3.1. Genome-Wide Identification and Characterization of the SNF7 Gene Family in Maize

In this study, the amino acid sequence of the conserved domain of AtSNF7 was used as the query template. Combined with the HMM specific to the SNF7 family, two rounds of genome-wide screening were performed via BLASTP and hmmsearch. All candidate genes were further verified for domain integrity using the Conserved Domain Database, which greatly improved the rigor and reliability of gene identification. Finally, 20 SNF7 family members in maize were identified, filling the research gap in systematic identification of the *SNF7* gene family in maize.

*SNF7* genes were first discovered in yeast and have been extensively characterized in yeast and mammals thereafter. Accordingly, Arabidopsis *SNF7* genes were named after their homologous counterparts in yeast and mammals, including SNF7, VPS20, VPS60, VPS24 and CHMP7. In the present study, the *SNF7* genes of rice and maize were not renamed, and their official gene locus IDs were adopted directly for labeling.

Clustering analysis based on sequence similarity classified the 20 ZmSNF7 proteins into three subfamilies, and members within the same subfamily shared highly consistent distribution patterns of conserved motifs. Nevertheless, no obvious subfamily-specific divergence was detected through gene structure characterization and three-dimensional protein structure prediction. These observations suggest that subfamily differentiation of the SNF7 family is mainly driven by sequence variations, rather than drastic remodeling of gene exon-intron structures or overall protein spatial conformations. Notably, three-dimensional structural prediction revealed that all ZmSNF7 proteins contain two conserved long α-helices in their central regions, further verifying the high evolutionary conservation of the core SNF7 domain. In contrast, the N- and C-terminal sequences exhibited substantial divergence across family members. Such terminal sequence variation is generally linked to protein functional specialization, implying that distinct *ZmSNF7* paralogs have undergone functional differentiation to satisfy diverse physiological demands during maize growth, development and stress responses.

Chromosomal localization analysis indicated that the 20 *ZmSNF7* genes were scattered across nine maize chromosomes without forming tandem duplication clusters. This genomic distribution pattern implies that each family member executes biological functions via independent transcriptional regulation, which alleviates functional redundancy within the gene family and provides a genomic structural basis for functional divergence of paralogs during evolution.

### 3.2. Evolutionary Specificity and Functional Divergence of ZmSNF7

Within the *ZmSNF7* gene family, the gene *Zm00001eb171860* exhibits distinctive multi-dimensional molecular features that distinguish it from all other paralogs, rendering it an evolutionarily unique case in this family. Genome-wide collinearity analysis revealed that this gene is the sole family member without detectable collinear orthologous copies in the genomes of maize inbred line Mo17 and teosinte SK. At the sequence level, it retains only two out of the ten core conserved motifs of the entire gene family. All other ZmSNF7 proteins possess a single canonical SNF7 conserved domain, whereas this protein additionally harbors a DUF1639 superfamily domain of unknown function. Meanwhile, its three-dimensional spatial conformation shares no significant similarity with other ZmSNF7 proteins.

Transcriptomic profiling demonstrated that this gene maintains extremely low transcript abundance across all maize tissues and organs, implying that it barely participates in regular maize growth and development. PPI prediction indicated that this protein is the only family member incapable of interacting with other *ZmSNF7* paralogs, failing to integrate into the intrinsic interaction network and breaking away from the canonical regulatory framework of the *SNF7* family. Cross-species conservation analysis further proved that this gene lacks the typical high sequence conservation characteristic of eukaryotic *SNF7* homologs, confirming that its evolutionary trajectory has diverged drastically from the rest of the family.

Taken together, the origin of this gene may be attributed to a chimeric gene-formation event, or it may represent a highly diverged or poorly annotated genomic locus.

### 3.3. Functional Potential of the ZmSNF7 Gene Family in Response to Abiotic and Biotic Stresses

Accumulating evidence has demonstrated that plant ESCRT components not only extensively modulate normal plant growth and developmental processes, but also play indispensable regulatory roles in plant responses to both biotic and abiotic stresses [29].

Cis-acting elements are core upstream regulatory elements governing gene transcription. Their types, abundance and distribution patterns can directly reflect the potential signaling pathways and biological functions of the corresponding genes. In this study, a large set of cis-acting elements related to hormone response, stress response, tissue-specific expression and growth/development were identified in the promoter regions of 20 *ZmSNF7* genes. Combined with the previous analysis of protein structural conservation and functional differentiation potential of this family, these findings provide critical clues for systematically deciphering the transcriptional regulatory mechanism of the *ZmSNF7* family. Notably, abscisic acid (ABA)- and methyl jasmonate (MeJA)-responsive elements are widely distributed in the promoters of most *ZmSNF7* genes, suggesting that ZmSNF7 members are potentially subject to ABA- and MeJA-dependent transcriptional regulation. In addition, the detection of abiotic stress-responsive elements (e.g., drought and low temperature) and tissue-specific elements (e.g., root- and seed-specific elements) further expands the functional scope of the *ZmSNF7* family. This is consistent with the conserved function of the plant SNF7 family in stress response, and also suggests remarkable spatiotemporal expression specificity among *ZmSNF7* members. Moreover, the presence of elements associated with cell cycle and circadian rhythm indicates that this gene family is also deeply involved in the regulation of basal growth and development in maize. It is speculated that *ZmSNF7* members have undergone functional specialization through the dual mechanism of protein structural divergence coupled with specific distribution of promoter regulatory elements: some members are mainly responsible for stress response, some focus on developmental regulation, and others may perform both biological functions. The exact pattern of functional differentiation remains to be verified by further experimental evidence.

Systematic mining of multiple transcriptome datasets provides more direct and robust transcript-level evidence for the stress-tolerant function of the *ZmSNF7* family. Expression analysis revealed that multiple *ZmSNF7* members exhibited significant differential expression under abiotic stress (drought) and three types of biotic stresses, including RBSDV infection, *C. graminicola* infection and ACB feeding.

Overall, the expression patterns of *ZmSNF7* genes quantified via qRT-PCR were largely concordant with the profiles retrieved from public transcriptome datasets, corroborating the reliability of our transcriptome-based expression profiling. Nevertheless, minor discrepancies in fold-change magnitudes were detected for a small subset of genes. These discrepancies can be primarily attributed to inherent differences in experimental systems between our validation assays and the original transcriptome studies. First, drought stress was mimicked by 10% PEG 8000-mediated osmotic stress in our experiments, as opposed to the natural soil drought treatments employed in the source datasets. Second, the Asian corn borer (ACB) bioassay was conducted using the B73 inbred line, which differs genotypically from the maize germplasms utilized in the original transcriptome study.

The responsive expression profiles displayed obvious member specificity under different stress conditions. These expression patterns are mutually corroborated the predictions based on promoter cis-elements, such as drought stress responsiveness and involvement in phytohormone signaling pathways. Collectively, evidence from both transcriptional regulatory prediction and stress expression analysis jointly supports the broad involvement of the *ZmSNF7* gene family in multiple stress responses in maize, and also provides a theoretical basis and data support for the application potential of this gene family in maize stress tolerance genetic improvement.

### 3.4. Candidate-Gene Application Potential of ZmSNF7 Family in Stress Responses

In this study, through cis-acting element analysis and systematic mining of multiple transcriptome datasets, we found that several *ZmSNF7* members exhibit broad-spectrum responsiveness to multiple stresses. Among them, *Zm00001eb126990* was downregulated under drought stress but significantly upregulated upon viral infection and insect herbivory, indicating that a single family member can integrate both biotic and abiotic stress signals and serves as a key regulatory node in the maize stress cross-talk network. Compared with most gene families that specifically respond to a single stress type, the *ZmSNF7* family covers four typical field stresses simultaneously: drought, viral infection, fungal pathogen infection, and insect herbivory. Meanwhile, distinct functional differentiation and expression divergence exist among *ZmSNF7* members. Through functional division and cooperative regulation, they form a multi-layered and temporally ordered regulatory network, ensuring coordinated stress responses and homeostasis maintenance in maize under complex field stresses (e.g., drought combined with pest and disease damage).

Based on the expression-profiling results in this study, three stress-responsive candidate genes (*Zm00001eb126990*, *Zm00001eb045910* and *Zm00001eb164660*) were identified for further functional validation. Subsequent molecular biological approaches, such as genetic transformation, can be adopted to systematically characterize these candidates and dissect their molecular mechanisms underlying maize stress responses. Once their biological functions are confirmed, these genes may serve as valuable genetic resources for stress-tolerance genetic improvement and breeding practice in maize.

This study still presents certain limitations in candidate gene exploration and application evaluation. The public transcriptome datasets were collected from independent studies with diverse maize genetic backgrounds and different plant growth stages, which may compromise the accuracy and universality of the identified stress-responsive expression patterns. Therefore, the stress adaptation functions and regulatory characteristics of the three candidate *ZmSNF7* genes require further validation through targeted functional experiments. Further investigation of their precise regulatory mechanisms in response to diverse stresses will clarify their application potential in multi-stress tolerance breeding, thereby providing a solid theoretical foundation for the genetic improvement of maize stress resistance.

## 4. Materials and Methods

### 4.1. Identification of the SNF7 Gene Family

The hidden Markov model (HMM) profile of SNF7 (Pfam accession: PF03357) was downloaded from the Pfam database [30], a member database of the InterPro resource (https://www.ebi.ac.uk/interpro/ (accessed on 6 March 2026)). Homology searches against the proteomes of maize, rice, and *Arabidopsis thaliana* were performed using HMMER v3.1 (https://www.ebi.ac.uk/Tools/hmmer/ (accessed on 6 March 2026)) [31] with an E-value cutoff of 0.01. Concurrently, BLASTP (https://blast.ncbi.nlm.nih.gov/Blast.cgi (accessed on 9 March 2026)) searches were conducted against the protein databases of the three species from Ensembl Plants (https://plants.ensembl.org/index.html (accessed on 9 April 2026)) using the conserved domain sequence of SNF7, with an E-value cutoff set to 1.0. All candidate *SNF7*-like genes were deduplicated and validated for the presence of the conserved Pfam domain, to finalize the complete member list of the *SNF7* gene family in maize, rice, and *Arabidopsis thaliana*.

The sequence length, molecular weight, isoelectric point (pI), grand average of hydropathicity (GRAVY), and instability index of SNF7 proteins were calculated using the ProtParam tool on ExPASy (https://web.expasy.org/protparam/ (accessed on 17 April 2026)) with default parameters, and summarized in a table. Signal peptide prediction was performed using SignalP-6.0 (https://services.healthtech.dtu.dk/services/SignalP-6.0/ (accessed on 17 April 2026)), with the organism group set to Eukarya. Subcellular localization prediction was conducted using the TargetP-2.0 online server (https://services.healthtech.dtu.dk/services/TargetP-2.0/ (accessed on 17 April 2026)), and plant was selected as the organism.

### 4.2. Phylogenetic Analysis of SNF7s

The full-length amino acid sequences of all SNF7 family members from *Zea mays*, *Oryza sativa* and *Arabidopsis thaliana* were downloaded from the Ensemble Plants database. Multiple sequence alignment was performed using ClustalW implemented in MEGA 11 (v11.0.11) [32]. The Neighbor-Joining phylogenetic tree was constructed with 1000 bootstrap replicates to explore the phylogenetic relationships among *ZmSNF7s*, *OsSNF7s*, and *AtSNF7s*, and all SNF7 members were further classified according to the tree topology. Subsequently, the phylogenetic tree was visualized and annotated using the online tool iTOL v5 (https://itol.embl.de/ (accessed on 16 April 2026).) [33].

### 4.3. Gene Structure, Conserved Sequence and Three-Dimensional Protein Structure Analyses

The version 5 genome annotation files of the maize inbred line B73 were downloaded from MaizeGDB (https://maizegdb.org/ (accessed on 15 April 2026)) for gene structure analysis of *ZmSNF7s*. Conserved Pfam domains were identified using the Batch CD-Search web server (http://52.37.121.124/Structure/bwrpsb/bwrpsb.cgi (accessed on 15 April 2026)), with an E-value cutoff of 0.01 against the Pfam-19638 PSSMs database Conserved motifs were identified using the MEME web server (https://meme-suite.org/meme/tools/meme (accessed on 15 April 2026)) [34], with the maximum number of motifs set to 10. Data visualization and graphical plotting were conducted using TBtools (v2.472) [35].

Three-dimensional structural models of ZmSNF7 proteins were generated using AlphaFold2 implemented in the Tamarind Bio online platform (https://app.tamarind.bio/app (accessed on 5 April 2026).), and all prediction procedures were run with default parameters [36].

### 4.4. Analysis of Regulatory Elements

The 2000 bp sequences upstream of the ATG start codon of *ZmSNF7* genes were extracted as promoter sequences. Cis-acting regulatory elements within these promoter regions were predicted using the PlantCARE online server (https://bioinformatics.psb.ugent.be/webtools/plantcare/html/ (accessed on 21 April 2026)) [37]. The functional annotation data were sorted and standardized using uniform terminology, and consistent annotations were assigned to cis-elements of the same type. The occurrence frequency of each cis-element in the promoter of each gene was calculated, and cis-element distribution diagrams and heatmaps for *ZmSNF7* genes were constructed using TBtools.

### 4.5. Chromosomal Localization, Collinearity Analysis and Ka/Ks Calculation

Genome sequences and gene annotation files of the maize inbred lines B73 and Mo17 were downloaded from MaizeGDB, and the relevant genomic data of teosinte SK were retrieved from NCBI (https://www.ncbi.nlm.nih.gov/ (accessed on 28 March 2026)). Genomic sequence alignment was conducted using the One-Step MCScanX tool [38] integrated in TBtools, with a BLASTP E-value cutoff of 1e-10. On this basis, the chromosomal distribution map of *ZmSNF7* family members and intraspecific collinearity Circos plots were visualized.

Collinearity among B73, Mo17 and teosinte SK were further analyzed using the Python library JCVI (v1.6.5) [39]. The genomic regions covering five upstream and five downstream flanking genes of each *ZmSNF7* family member were extracted to construct interspecific collinearity graphs.

Paralogous pairs of the *ZmSNF7* gene family were identified via all-vs-all BLASTP alignment of protein sequences, with screening thresholds of E-value ≤ 1 × 10^−5^, sequence identity ≥ 50%, and alignment coverage ≥ 70%. The nonsynonymous substitution rate (Ka), synonymous substitution rate (Ks), and Ka/Ks ratio for each paralogous pair were calculated using the Simple Ka/Ks Calculator in TBtools with default parameters. Full-length CDS were extracted from the maize B73 reference genome, followed by pairwise CDS alignment with the built-in MAFFT algorithm and substitution rate estimation under the Yang-Nielsen (YN) model.

### 4.6. Protein Interaction Prediction and Functional Annotation Evaluation

The ZmSNF7 protein sequences were analyzed on the STRING web platform (https://cn.string-db.org/ (accessed on 28 April 2026)) using Maize as a reference. Including the prediction of internal interactions among *ZmSNF7* family members and the interaction relationships between ZmSNF7 proteins and other proteins.

Functional enrichment of GO terms for the *ZmSNF7* gene family in maize was performed using the online tool agriGO v2.0 (https://systemsbiology.cau.edu.cn/agriGOv2/ (accessed on 5 June 2026)) [40] with all default parameters. GO terms with adjusted *p*-value < 0.01 were retained for subsequent visualization. Bubble plots illustrating GO enrichment results were generated via the R (v4.5.1) programming language.

### 4.7. Expression Heatmap of Maize ZmSNF7 Family Genes Across Different Tissues

The tissue expression patterns of *ZmSNF7* genes were analyzed based on publicly available transcriptome data from a previous published study [41]. The original study integrated seven independent transcriptome datasets comprising a total of 422 plant tissue samples. Briefly, detailed sample background information and metadata were summarized in Dataset S1, while the raw expression levels of functional genes were listed in Dataset S2. In the present study, these two supplementary datasets were downloaded and screened. The expression data of 20 identified *ZmSNF7* family members were specifically extracted for subsequent expression pattern analysis. Finally, a tissue-specific expression heatmap of the *ZmSNF7* gene family was visualized and generated using TBtools software.

### 4.8. Transcriptome Analysis of ZmSNF7 Gene Family

Transcriptomic data of maize subjected to abiotic and biotic stresses were downloaded from the NCBI Sequence Read Archive (SRA) database (https://www.ncbi.nlm.nih.gov/sra (accessed on 18 May 2026)), with the corresponding project numbers as follows: drought (PRJNA1404907), RBSDV (PRJNA760309), *C. graminicola* (PRJNA868217) and ACB (PRJNA761215) [42].

Quality control and filtering of sequencing reads were performed using Sickle (v1.33) [43]. The clean reads were aligned to the maize B73 reference genome with HISAT2 (v2.2.2) [44]. The generated SAM files were converted into sorted BAM files using samtools (v1.19.2) [45]. Gene count extraction and FPKM (Fragments Per Kilobase of transcript per Million mapped reads) calculation were performed using featureCounts (v2.1.1) [46] and StringTie (v2.2.3) [47]. Differentially expressed gene (DEG) analysis was conducted using DESeq2 (v1.52.0) [48]. Genes with an average count less than 10 across all samples were filtered out prior to differential analysis. Genes with |log_2_ (fold change)| ≥ 1 and adjusted *p*-value (padj) < 0.05 were identified as differentially expressed genes. All data visualization was performed with GraphPad Prism 8.0.2.

### 4.9. qRT-PCR Assay for ZmSNF7s Under Stress Conditions

Seeds of maize inbred line B73 were germinated and grown hydroponically on germination paper. At the two-leaf stage, seedlings were subjected to stress treatments. For drought stress, seedlings were treated with 10% (*w*/*v*) PEG 8000 (Sigma-Aldrich, St. Louis, MO, USA), whereas control seedlings were maintained in pure water. Leaves were harvested at 1 and 2 days after treatment (DAT). For *Fusarium verticillioides* infection, seedling root tips were inoculated as previously described [49], and leaves were collected at 1 and 2 DAT. For herbivory stress, third-instar larvae of the Asian corn borer were allowed to feed on maize seedlings, and leaves were sampled at 1 and 2 h after infestation, with untreated seedlings serving as the control. Each sample consisted of pooled leaves from five seedlings, with three biological replicates per time point.

Total RNA was extracted from seedling leaves using Trizol reagent (Coolaber, Beijing, China). Reverse transcription was performed using the PrimeScript™ RT reagent Kit with gDNA Eraser (TAKARA, Shiga, Japan). qPCR was carried out using the Taq Pro Universal SYBR qPCR Master Mix (Vazyme, Nanjing, China). Relative gene expression levels were calculated using the 2^(−ΔΔCt)^ method. All procedures were performed according to the manufacturers’ instructions. Primer sequences used for qPCR are listed in Table S6.

## 5. Conclusions

In this study, a total of 20 *SNF7* genes were identified with high confidence across the maize genome. Phylogenetic clustering classified these genes into three distinct subfamilies, whose members shared highly conserved motif compositions. Combined analyses of cis-acting regulatory elements in promoter regions, PPI networks and GO annotations revealed that ZmSNF7 proteins interact with the ESCRT complex to mediate intracellular protein sorting and degradation, thereby regulating plant hormone signaling and responses to diverse environmental stimuli. Transcriptomic data derived from RNA-seq of 422 maize samples covering various tissues and organs demonstrated that most *ZmSNF7* genes exhibited moderate expression levels throughout growth and developmental stages of maize. Further mining and reanalysis of transcriptome datasets under biotic and abiotic stresses indicated that the *ZmSNF7* gene family responds to drought stress, RBSDV infection, *C. graminicola* challenge, and ACB herbivory. This study completes the genome-wide identification of *SNF7* genes in maize, which advances our understanding of the genetic basis underlying maize growth, development and stress resistance traits.

## Figures and Tables

**Table 1:** Physicochemical characteristics of the identified ZmSNF7 proteins in maize.

Gene ID	Amino Acid	Molecular Weight (kDa)	Theoretical pI	Instability Index	GRAVY
Zm00001eb162120	222	24.58	4.83	44.04	−0.61
Zm00001eb171860	201	22.19	9.64	45.38	−0.764
Zm00001eb061610	222	24.73	4.83	45.77	−0.623
Zm00001eb386640	226	24.96	4.85	46.82	−0.567
Zm00001eb321740	100	11.58	9.67	37.33	−0.737
Zm00001eb195640	223	24.99	4.84	61.49	−0.796
Zm00001eb358220	222	24.57	4.83	46.54	−0.636
Zm00001eb126990	230	25.86	4.76	52.59	-0.822
Zm00001eb267590	228	25.65	4.7	43.47	−0.826
Zm00001eb129510	411	46.56	5.34	53.67	−0.24
Zm00001eb183830	266	29.73	4.63	44.45	−0.425
Zm00001eb246180	231	25.9	4.75	48.21	−0.632
Zm00001eb218770	229	25.2	9.32	48.78	−0.62
Zm00001eb045910	212	23.36	6.78	49.43	−0.692
Zm00001eb303830	223	25.01	5.36	49.57	−0.613
Zm00001eb094970	223	25.07	5.36	53.39	−0.625
Zm00001eb164660	224	25	5.74	48.72	−0.543
Zm00001eb387090	204	22.82	7.77	32.73	−0.621
Zm00001eb000670	228	25.79	5.57	50.05	−0.771
Zm00001eb321340	229	25.98	5.42	54.09	−0.809

Notes: kDa, kilodalton; pI, theoretical isoelectric point; GRAVY, grand average of hydropathicity.

## Data Availability

All data used in this study are available in the Supplementary Materials or from NCBI.

## Supplementary Materials

The following supporting information can be downloaded at https://www.mdpi.com/article/10.3390/ijms27187985/s1.

## Abbreviations

The following abbreviations are used in this manuscript:

SNF7	Sucrose non-fermenting protein 7
ESCRT	Endosomal sorting complex required for transport
GO	Gene ontology
RBSDV	Rice black-streaked dwarf virus
ACB	Asian corn borer
ILVs	Intraluminal vesicles
MVBs	Multivesicular bodies
PVCs	Prevacuolar compartments
PM	Plasma membrane
VPS	Vacuolar protein sorting
VTA	VPS twenty-associated
ATP	Adenosine triphosphate
HMM	Hidden Markov model
GRAVY	Grand average of hydropathicity
pI	Isoelectric point
TPM	Transcripts Per Million
FPKM	Fragments Per Kilobase of transcript per Million mapped reads
PPI	Protein–protein interaction
CHMP	Charged multivesicular body protein
SKD1	Suppressor of K^+^ transport growth defect 1
hpi	Hours post-inoculation
MVB	Multivesicular body
ABA	Abscisic acid
MeJA	Methyl jasmonate
