# Supplementary material for: Genome-Wide Identification of SNF7 Gene Family in Maize and Potential Roles in Response to Abiotic and Biotic Stress
Source: Int J Mol Sci. 2026 Sep 8;27(18):7985. doi: 10.3390/ijms27187985 (PMC13606720; doi:10.3390/ijms27187985)
Supplement: Supplementary file 1 [file ijms-27-07985-s001.zip › ijms-4437893-supplementary.pdf]

Table S1. Physicochemical characteristics of the identified SNF7 proteins in rice and *Arabidopsis*

Gene ID	Amino acid lengh (aa)	Molecular weight (kDa)	Theoretical pI	Instability index	GRAVY
Os12g0121400	216	24.16	4.91	44.31	-0.696
Os11g0703400	224	24.86	5.73	49.82	-0.489
Os11g0123500	216	24.10	4.91	48.47	-0.678
Os09g0267600	299	32.85	5.23	57.50	-0.653
Os03g0108400	231	26.12	5.26	50.06	-0.843
Os07g0490700	97	11.22	9.48	40.06	-0.918
Os10g0476400	212	23.42	6.78	51.06	-0.714
Os05g0102900	232	26.12	4.69	46.58	-0.907
Os07g0236800	225	25.15	5.52	48.43	-0.642
Os06g0608500	220	24.23	4.84	46.26	-0.511
Os01g0103000	232	26.13	4.81	51.04	-0.826
Os02g0596500	233	26.00	4.68	47.50	-0.640
Os06g0643300	205	22.84	7.77	33.34	-0.570
Os01g0286000	419	47.65	5.14	52.81	-0.377
Os03g0639800	229	25.40	9.22	46.37	-0.705
AT1G03950	210	23.03	6.87	47.10	-0.685
AT3G45000	200	22.04	4.92	52.83	-0.330
AT3G62080	462	51.92	5.01	42.49	-0.373
AT5G44560	222	24.30	9.54	53.17	-0.572
AT5G04850	272	30.31	4.77	41.25	-0.622
AT1G17730	203	22.76	6.66	45.28	-0.665
AT2G06530	225	25.29	5.54	51.86	-0.674
AT2G19830	213	24.01	4.78	45.32	-0.733
AT5G22950	229	26.00	5.58	57.04	-0.665
AT4G29160	219	24.33	4.89	53.97	-0.656
AT5G09260	216	24.61	4.78	54.44	-0.666
AT3G10640	235	26.17	4.78	46.74	-0.863
AT1G73030	203	22.70	6.66	42.80	-0.650
AT5G63880	243	27.67	4.64	50.70	-0.600

Table S2. Prediction of signal peptides of ZmSNF7 protein family in maize using SignalP-6.0

Gene ID	Prediction	OTHER	SP(Sec/SPI)	CS Position
Zm00001eb358220	OTHER	1	0	NA
Zm00001eb162120	OTHER	1	0	NA
Zm00001eb171860	OTHER	1	0	NA
Zm00001eb126990	OTHER	1	0	NA
Zm00001eb321740	OTHER	1	0.000001	NA
Zm00001eb218770	OTHER	1	0	NA
Zm00001eb045910	OTHER	1	0	NA
Zm00001eb164660	OTHER	1	0	NA
Zm00001eb267590	OTHER	1	0	NA
Zm00001eb000670	OTHER	1	0	NA
Zm00001eb303830	OTHER	1	0.000001	NA
Zm00001eb183830	OTHER	1	0	NA
Zm00001eb195640	OTHER	1	0.000001	NA
Zm00001eb094970	OTHER	1	0.000001	NA
Zm00001eb386640	OTHER	1	0	NA
Zm00001eb061610	OTHER	1	0.000001	NA
Zm00001eb129510	OTHER	1	0	NA
Zm00001eb246180	OTHER	1	0	NA
Zm00001eb321340	OTHER	1	0	NA
Zm00001eb387090	OTHER	1	0	NA

note: SP, Signal Peptide; CS, Cleavage Site; SPI, Signal Peptidase I; NA, not applicable. Prediction results were obtained from SignalP-6.0 with Eukarya model.

Table S3. Subcellular localization prediction of ZmSNF7 protein family in maize using TargetP-2.0

Gene ID	Prediction	OTHER	SP	mTP	cTP	lTP	CS Position
Zm00001eb358220	OTHER	0.9993	0.000008	0.000727	0.000001	0	NA
Zm00001eb162120	OTHER	0.9985	0.00001	0.001472	0.000001	0	NA
Zm00001eb171860	OTHER	0.9822	0.000862	0.01024	0.00665	0.000047	NA
Zm00001eb126990	OTHER	0.9999	0.000012	0.000044	0	0	NA
Zm00001eb321740	OTHER	0.9866	0.000114	0.013177	0.000002	0.000084	NA
Zm00001eb218770	OTHER	0.9995	0.000013	0.000483	0.000001	0.000001	NA
Zm00001eb045910	OTHER	0.9999	0.000015	0.000098	0.000005	0.000003	NA
Zm00001eb164660	OTHER	0.9998	0.000017	0.000208	0.000002	0	NA
Zm00001eb267590	OTHER	1	0.000013	0.000034	0	0	NA
Zm00001eb000670	OTHER	1	0.00002	0.000025	0.000002	0.000002	NA
Zm00001eb303830	OTHER	0.9991	0.000009	0.000936	0.000002	0.000001	NA
Zm00001eb183830	OTHER	0.9997	0.000061	0.00028	0.000005	0.000001	NA
Zm00001eb195640	OTHER	0.9969	0.000002	0.003129	0	0	NA
Zm00001eb094970	OTHER	0.999	0.000009	0.000964	0.000002	0.000001	NA
Zm00001eb386640	OTHER	0.9996	0.000016	0.000408	0	0	NA
Zm00001eb061610	OTHER	0.9988	0.00001	0.001145	0.000001	0	NA
Zm00001eb129510	OTHER	1	0.000047	0.000003	0.000001	0	NA
Zm00001eb246180	OTHER	0.9993	0.000072	0.00064	0.00001	0.000002	NA
Zm00001eb321340	OTHER	1	0.000002	0.000026	0.000001	0.000001	NA
Zm00001eb387090	OTHER	1	0.000029	0.000014	0	0	NA

Note: SP, signal peptide; mTP, mitochondrial transit peptide; cTP, chloroplast transit peptide; lTP, lysosomal transit peptide; CS Position, cleavage site position; NA, not applicable.

Table S4. STRING-predicted interacting partners of maize ZmSNF7 (B4FU00_MAIZE)

Protein ID	Protein Description	Score
B8A2W9_MAIZE	Protein suppressor of K (+) transport growth defect 1; Belongs to the AAA ATPase family.	0.958
Pco062079a	Charged multivesicular body protein 5.	0.9
A0A1D6LXR8	LYST-interacting protein 5-like protein.	0.772
Umc2756	LYST-interacting protein 5-like protein.	0.772
A0A1D6NZK8	LYST-interacting protein 5-like protein.	0.772
A0A1D6FE31	Vacuolar sorting protein 4b; Belongs to the AAA ATPase family.	0.601
A0A1D6NWJ2	Uncharacterized protein.	0.601
A0A1D6LQX2	ESCRT-related protein CHMP1B.	0.497
A0A1D6LQX3	ESCRT-related protein CHMP1B.	0.497
Sal1	Supernumerary aleurone1.	0.497
A0A1D6IBE8	Vacuolar protein sorting-associated protein 24 homolog 1.	0.431
Umc2748	Vacuolar protein sorting-associated protein 24 homolog 1.	0.431
A0A1D6FZB5	Vacuolar protein sorting-associated protein 32 homolog 1.	0.4
A0A1D6L8F6	Vacuolar protein sorting-associated protein 32 homolog 1.	0.4
A0A1D6NMB1	Vacuolar protein sorting-associated protein 32 homolog 1.	0.4
A0A1D6P3C0	Charged multivesicular body protein 4b.	0.4
A0A1D6PV38	Vacuolar protein sorting-associated protein 32 homolog 1.	0.4
A0A1D6QGS9	Vacuolar protein sorting-associated protein 32 homolog 1.	0.4
B6U8I2_MAIZE	Vacuolar protein sorting-associated protein 32 homolog 1.	0.4
K7WAK3_MAIZE	Uncharacterized protein.	0.4

Table S5. Detailed differential expression statistics of *ZmSNF7* genes under various stress conditions.

GeneID	Treatment comparison	log2FC	P-value	padj
Zm00001eb000670	Drought_D1	NA	NA	NA
Zm00001eb045910	Drought_D1	0.18	2.09E-02	9.04E-02
Zm00001eb061610	Drought_D1	NA	NA	NA
Zm00001eb094970	Drought_D1	-0.61	1.83E-01	4.02E-01
Zm00001eb126990	Drought_D1	-0.85	4.47E-01	5.73E-01
Zm00001eb129510	Drought_D1	NA	NA	NA
Zm00001eb162120	Drought_D1	NA	NA	NA
Zm00001eb164660	Drought_D1	1.11	3.22E-42	4.19E-41
Zm00001eb171860	Drought_D1	NA	NA	NA
Zm00001eb183830	Drought_D1	NA	NA	NA
Zm00001eb195640	Drought_D1	-0.22	5.73E-01	5.73E-01
Zm00001eb218770	Drought_D1	NA	NA	NA
Zm00001eb246180	Drought_D1	-0.63	5.61E-01	5.73E-01
Zm00001eb267590	Drought_D1	0.30	1.52E-01	4.02E-01
Zm00001eb303830	Drought_D1	-0.91	2.87E-27	1.87E-26
Zm00001eb321340	Drought_D1	-0.12	2.16E-01	4.02E-01
Zm00001eb321740	Drought_D1	-0.88	5.00E-01	5.73E-01
Zm00001eb358220	Drought_D1	1.72	2.12E-01	4.02E-01
Zm00001eb386640	Drought_D1	0.58	5.04E-01	5.73E-01
Zm00001eb387090	Drought_D1	0.08	4.15E-01	5.73E-01
Zm00001eb000670	Drought_D4	NA	NA	NA
Zm00001eb045910	Drought_D4	-0.20	1.11E-02	3.61E-02
Zm00001eb061610	Drought_D4	NA	NA	NA
Zm00001eb094970	Drought_D4	-0.22	6.30E-01	7.83E-01
Zm00001eb126990	Drought_D4	-0.48	6.63E-01	7.83E-01
Zm00001eb129510	Drought_D4	NA	NA	NA
Zm00001eb162120	Drought_D4	NA	NA	NA
Zm00001eb164660	Drought_D4	0.65	3.08E-15	4.01E-14
Zm00001eb171860	Drought_D4	NA	NA	NA
Zm00001eb183830	Drought_D4	NA	NA	NA
Zm00001eb195640	Drought_D4	-0.02	9.50E-01	9.50E-01
Zm00001eb218770	Drought_D4	NA	NA	NA
Zm00001eb246180	Drought_D4	2.47	7.85E-03	3.40E-02
Zm00001eb267590	Drought_D4	0.13	5.49E-01	7.83E-01
Zm00001eb303830	Drought_D4	0.12	1.37E-01	3.55E-01
Zm00001eb321340	Drought_D4	-0.08	3.95E-01	6.81E-01
Zm00001eb321740	Drought_D4	1.10	3.54E-01	6.81E-01
Zm00001eb358220	Drought_D4	1.14	4.19E-01	6.81E-01
Zm00001eb386640	Drought_D4	0.19	8.34E-01	9.04E-01
Zm00001eb387090	Drought_D4	-0.44	2.41E-06	1.56E-05
Zm00001eb000670	RBSDV	NA	NA	NA

Zm00001eb045910	RBSDV	0.28	1.59E-02	3.19E-02
Zm00001eb061610	RBSDV	NA	NA	NA
Zm00001eb094970	RBSDV	-0.54	4.23E-02	7.25E-02
Zm00001eb126990	RBSDV	1.68	4.24E-03	1.02E-02
Zm00001eb129510	RBSDV	NA	NA	NA
Zm00001eb162120	RBSDV	NA	NA	NA
Zm00001eb164660	RBSDV	0.07	6.03E-01	8.03E-01
Zm00001eb171860	RBSDV	NA	NA	NA
Zm00001eb183830	RBSDV	NA	NA	NA
Zm00001eb195640	RBSDV	-0.04	8.99E-01	9.84E-01
Zm00001eb218770	RBSDV	NA	NA	NA
Zm00001eb246180	RBSDV	NA	NA	NA
Zm00001eb267590	RBSDV	-0.88	1.27E-08	1.53E-07
Zm00001eb303830	RBSDV	0.18	1.85E-01	2.78E-01
Zm00001eb321340	RBSDV	0.35	2.32E-03	9.29E-03
Zm00001eb321740	RBSDV	0.02	9.80E-01	9.84E-01
Zm00001eb358220	RBSDV	0.02	9.84E-01	9.84E-01
Zm00001eb386640	RBSDV	-1.03	2.39E-04	1.43E-03
Zm00001eb387090	RBSDV	-0.34	3.49E-03	1.02E-02
Zm00001eb000670	C. graminicola_24h	NA	NA	NA
Zm00001eb045910	C. graminicola_24h	-0.14	3.87E-01	4.25E-01
Zm00001eb061610	C. graminicola_24h	NA	NA	NA
Zm00001eb094970	C. graminicola_24h	-1.09	6.74E-06	3.71E-05
Zm00001eb126990	C. graminicola_24h	-1.14	2.46E-02	3.87E-02
Zm00001eb129510	C. graminicola_24h	NA	NA	NA
Zm00001eb162120	C. graminicola_24h	NA	NA	NA
Zm00001eb164660	C. graminicola_24h	-0.07	3.55E-01	4.25E-01
Zm00001eb171860	C. graminicola_24h	NA	NA	NA
Zm00001eb183830	C. graminicola_24h	NA	NA	NA
Zm00001eb195640	C. graminicola_24h	0.47	4.88E-02	6.71E-02
Zm00001eb218770	C. graminicola_24h	NA	NA	NA
Zm00001eb246180	C. graminicola_24h	NA	NA	NA
Zm00001eb267590	C. graminicola_24h	0.12	5.14E-01	5.14E-01
Zm00001eb303830	C. graminicola_24h	0.41	1.66E-05	6.10E-05
Zm00001eb321340	C. graminicola_24h	0.36	2.99E-04	8.22E-04
Zm00001eb321740	C. graminicola_24h	NA	NA	NA
Zm00001eb358220	C. graminicola_24h	2.03	1.30E-02	2.39E-02
Zm00001eb386640	C. graminicola_24h	-2.15	1.15E-02	2.39E-02
Zm00001eb387090	C. graminicola_24h	-0.78	6.04E-06	3.71E-05
Zm00001eb000670	C. graminicola_40h	NA	NA	NA
Zm00001eb045910	C. graminicola_40h	0.16	3.29E-01	4.53E-01
Zm00001eb061610	C. graminicola_40h	NA	NA	NA
Zm00001eb094970	C. graminicola_40h	0.31	1.69E-01	3.72E-01
Zm00001eb126990	C. graminicola_40h	1.66	1.00E-04	1.10E-03

Zm00001eb129510	C. graminicola_40h	NA	NA	NA
Zm00001eb162120	C. graminicola_40h	NA	NA	NA
Zm00001eb164660	C. graminicola_40h	-0.05	5.19E-01	5.85E-01
Zm00001eb171860	C. graminicola_40h	NA	NA	NA
Zm00001eb183830	C. graminicola_40h	NA	NA	NA
Zm00001eb195640	C. graminicola_40h	-0.15	5.32E-01	5.85E-01
Zm00001eb218770	C. graminicola_40h	NA	NA	NA
Zm00001eb246180	C. graminicola_40h	NA	NA	NA
Zm00001eb267590	C. graminicola_40h	0.39	3.49E-02	1.92E-01
Zm00001eb303830	C. graminicola_40h	-0.10	2.86E-01	4.49E-01
Zm00001eb321340	C. graminicola_40h	0.16	1.01E-01	2.77E-01
Zm00001eb321740	C. graminicola_40h	NA	NA	NA
Zm00001eb358220	C. graminicola_40h	1.60	5.51E-02	2.02E-01
Zm00001eb386640	C. graminicola_40h	0.76	2.76E-01	4.49E-01
Zm00001eb387090	C. graminicola_40h	0.07	6.93E-01	6.93E-01
Zm00001eb000670	C. graminicola_60h	NA	NA	NA
Zm00001eb045910	C. graminicola_60h	0.34	3.69E-02	5.80E-02
Zm00001eb061610	C. graminicola_60h	NA	NA	NA
Zm00001eb094970	C. graminicola_60h	0.28	2.16E-01	2.97E-01
Zm00001eb126990	C. graminicola_60h	0.98	2.38E-02	4.70E-02
Zm00001eb129510	C. graminicola_60h	NA	NA	NA
Zm00001eb162120	C. graminicola_60h	NA	NA	NA
Zm00001eb164660	C. graminicola_60h	-0.39	8.87E-07	9.76E-06
Zm00001eb171860	C. graminicola_60h	NA	NA	NA
Zm00001eb183830	C. graminicola_60h	NA	NA	NA
Zm00001eb195640	C. graminicola_60h	-0.94	2.59E-04	1.43E-03
Zm00001eb218770	C. graminicola_60h	NA	NA	NA
Zm00001eb246180	C. graminicola_60h	NA	NA	NA
Zm00001eb267590	C. graminicola_60h	0.49	7.86E-03	2.16E-02
Zm00001eb303830	C. graminicola_60h	-0.30	1.59E-03	5.83E-03
Zm00001eb321340	C. graminicola_60h	0.22	2.56E-02	4.70E-02
Zm00001eb321740	C. graminicola_60h	NA	NA	NA
Zm00001eb358220	C. graminicola_60h	0.21	8.12E-01	8.93E-01
Zm00001eb386640	C. graminicola_60h	0.48	4.94E-01	6.03E-01
Zm00001eb387090	C. graminicola_60h	0.00	9.89E-01	9.89E-01
Zm00001eb000670	C. graminicola_96h	NA	NA	NA
Zm00001eb045910	C. graminicola_96h	0.93	1.72E-08	1.89E-07
Zm00001eb061610	C. graminicola_96h	NA	NA	NA
Zm00001eb094970	C. graminicola_96h	-0.81	5.43E-04	1.19E-03
Zm00001eb126990	C. graminicola_96h	-3.24	6.56E-06	3.61E-05
Zm00001eb129510	C. graminicola_96h	NA	NA	NA
Zm00001eb162120	C. graminicola_96h	NA	NA	NA
Zm00001eb164660	C. graminicola_96h	0.17	3.07E-02	4.22E-02
Zm00001eb171860	C. graminicola_96h	NA	NA	NA

Zm00001eb183830	C. graminicola_96h	NA	NA	NA
Zm00001eb195640	C. graminicola_96h	-0.48	4.94E-02	6.03E-02
Zm00001eb218770	C. graminicola_96h	NA	NA	NA
Zm00001eb246180	C. graminicola_96h	NA	NA	NA
Zm00001eb267590	C. graminicola_96h	0.75	4.10E-05	1.13E-04
Zm00001eb303830	C. graminicola_96h	-0.10	2.88E-01	3.17E-01
Zm00001eb321340	C. graminicola_96h	0.42	2.57E-05	9.41E-05
Zm00001eb321740	C. graminicola_96h	NA	NA	NA
Zm00001eb358220	C. graminicola_96h	-0.18	8.43E-01	8.43E-01
Zm00001eb386640	C. graminicola_96h	1.93	4.02E-03	7.37E-03
Zm00001eb387090	C. graminicola_96h	-0.47	5.67E-03	8.91E-03
Zm00001eb000670	PHW03_ACB_2h	NA	NA	NA
Zm00001eb045910	PHW03_ACB_2h	-0.80	2.01E-05	1.21E-04
Zm00001eb061610	PHW03_ACB_2h	NA	NA	NA
Zm00001eb094970	PHW03_ACB_2h	0.57	4.10E-02	7.03E-02
Zm00001eb126990	PHW03_ACB_2h	1.52	2.38E-03	5.71E-03
Zm00001eb129510	PHW03_ACB_2h	NA	NA	NA
Zm00001eb162120	PHW03_ACB_2h	NA	NA	NA
Zm00001eb164660	PHW03_ACB_2h	-0.58	7.92E-04	2.38E-03
Zm00001eb171860	PHW03_ACB_2h	NA	NA	NA
Zm00001eb183830	PHW03_ACB_2h	NA	NA	NA
Zm00001eb195640	PHW03_ACB_2h	1.17	6.49E-04	2.38E-03
Zm00001eb218770	PHW03_ACB_2h	NA	NA	NA
Zm00001eb246180	PHW03_ACB_2h	NA	NA	NA
Zm00001eb267590	PHW03_ACB_2h	-0.49	1.04E-02	2.09E-02
Zm00001eb303830	PHW03_ACB_2h	0.05	7.84E-01	9.36E-01
Zm00001eb321340	PHW03_ACB_2h	0.03	6.83E-01	9.11E-01
Zm00001eb321740	PHW03_ACB_2h	0.63	3.62E-01	5.43E-01
Zm00001eb358220	PHW03_ACB_2h	0.13	8.58E-01	9.36E-01
Zm00001eb386640	PHW03_ACB_2h	-0.09	9.52E-01	9.52E-01
Zm00001eb387090	PHW03_ACB_2h	-0.48	1.24E-07	1.49E-06
Zm00001eb000670	LH162_ACB_2h	NA	NA	NA
Zm00001eb045910	LH162_ACB_2h	-0.40	1.77E-02	7.09E-02
Zm00001eb061610	LH162_ACB_2h	NA	NA	NA
Zm00001eb094970	LH162_ACB_2h	0.41	1.77E-01	2.36E-01
Zm00001eb126990	LH162_ACB_2h	0.44	2.35E-01	2.81E-01
Zm00001eb129510	LH162_ACB_2h	NA	NA	NA
Zm00001eb162120	LH162_ACB_2h	NA	NA	NA
Zm00001eb164660	LH162_ACB_2h	-0.60	1.61E-04	1.94E-03
Zm00001eb171860	LH162_ACB_2h	NA	NA	NA
Zm00001eb183830	LH162_ACB_2h	NA	NA	NA
Zm00001eb195640	LH162_ACB_2h	0.12	6.84E-01	6.84E-01
Zm00001eb218770	LH162_ACB_2h	NA	NA	NA
Zm00001eb246180	LH162_ACB_2h	NA	NA	NA

Zm00001eb267590	LH162_ACB_2h	-0.41	5.37E-02	1.35E-01
Zm00001eb303830	LH162_ACB_2h	0.28	5.61E-02	1.35E-01
Zm00001eb321340	LH162_ACB_2h	0.24	1.05E-01	2.11E-01
Zm00001eb321740	LH162_ACB_2h	1.07	1.51E-01	2.27E-01
Zm00001eb358220	LH162_ACB_2h	1.26	1.29E-01	2.21E-01
Zm00001eb386640	LH162_ACB_2h	-0.55	4.82E-01	5.26E-01
Zm00001eb387090	LH162_ACB_2h	-0.57	1.28E-03	7.65E-03

Note: NA indicates that samples with counts less than 10 were filtered out prior to differential expression analysis.

Table S6. qPCR primer sequences for 12 *ZmSNFs* genes

Gene ID	Forward primer (5′-3′)	Reverse primer (5′-3′)
Zm00001eb045910	CATTGCGTCATTACAGCAGGAG	TCAGCTGACGGGCTAGAATTTT
Zm00001eb094970	GCCAAAGATCTTATTCGCACCC	TCACTCACCATCTCCATCCTCT
Zm00001eb126990	CAATGAAGGCAGCAAACAAGGA	CCCATCAGTTCTTCCTCGTCAA
Zm00001eb164660	AAGAAGAAGACTCCTGCTGAGC	TTTTACAGCTCCCATCTGACCC
Zm00001eb195640	CCGCAATCCAGTGCCTAA	CCTTGACAGCAGATGACCC
Zm00001eb267590	GGACGCAACCGACAGAAT	TGCCCTTCGTACATCCTG
Zm00001eb303830	ACCCGGCATCAGATAACAAAGT	TGCCTGTTCATTTGAGCCATTG
Zm00001eb321340	GACATGGAGGAGGAAATCG	CTCGTCGTCATCAGCACC
Zm00001eb321740	CTTCAAGGCGAAAGAGGTG	GCTTGTTTCCCGACTTTGTG
Zm00001eb358220	GCCACTCTGGACAAGTTAA	ATTGGATAGCCGCTCTTT
Zm00001eb386640	TCGCTCGACAAGCTCAAC	TCCAAGCTGCTCAATCTG
Zm00001eb387090	GCATCTACGCCGAGAACGC	TGGACTTGCCGATGACCTG

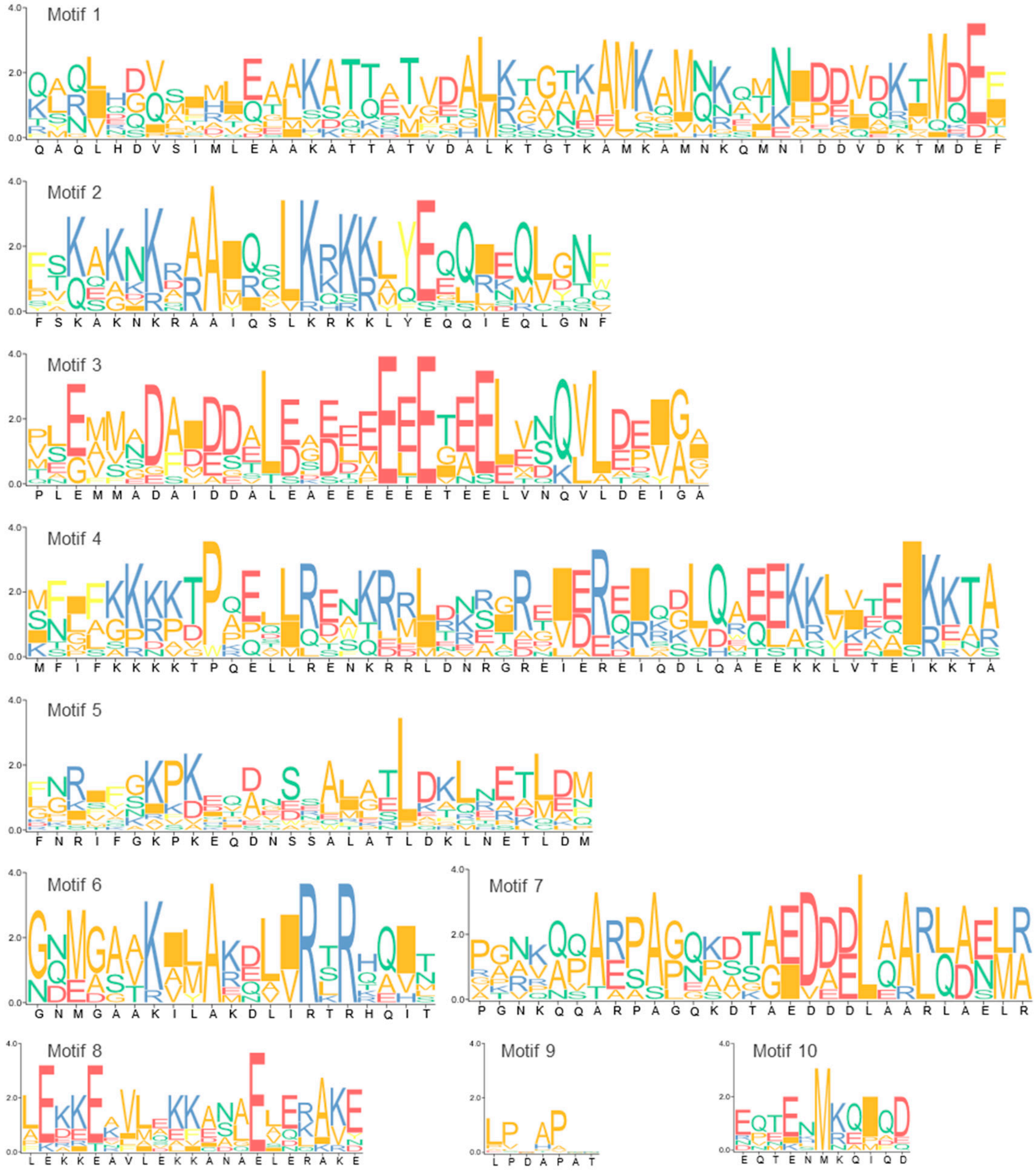

Figure S1. Sequence logo of 10 predicted conserved motifs in the ZmSNF7 proteins.

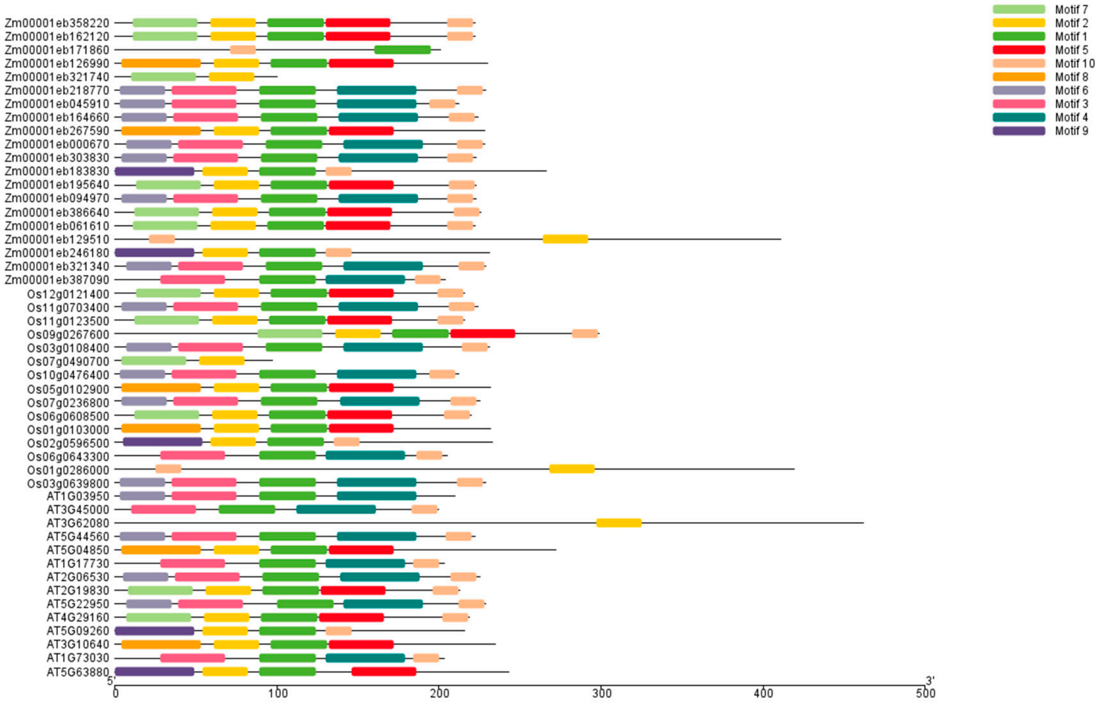

Figure S2. Conserved-motif analysis of SNF7 gene family members in *Zea mays*, *Oryza sativa*, and *Arabidopsis thaliana*.

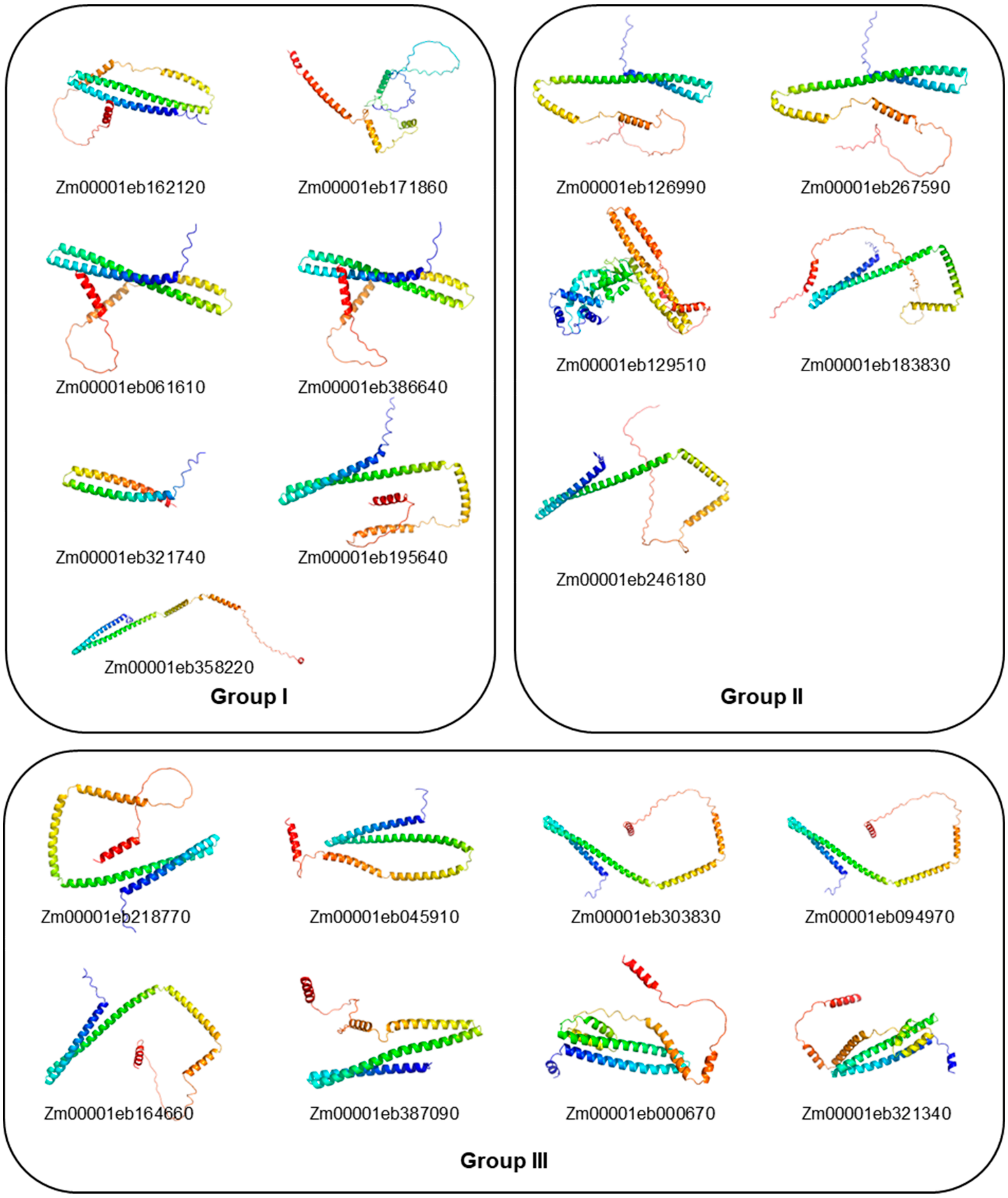

Figure S3. Three-dimensional structure modeling of ZmSNF7 proteins. Each protein structure is colored using a rainbow gradient scheme, progressing from the N-terminus (blue) to the C-terminus (red).

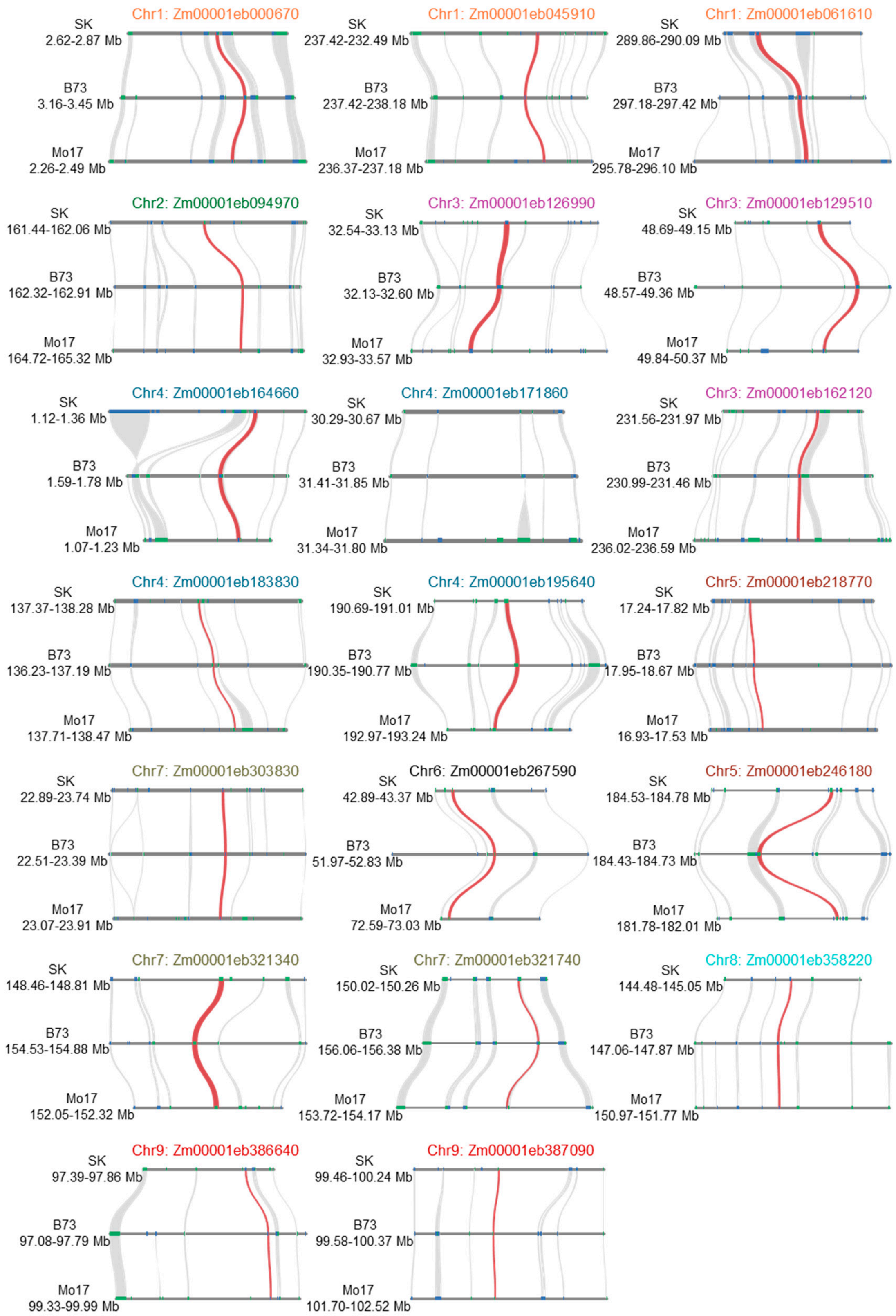

Figure S4. Collinearity Analysis of the *ZmSNF7* Gene Family Among Maize Inbred Lines B73, Mo17, and Teosinte Accession SK.

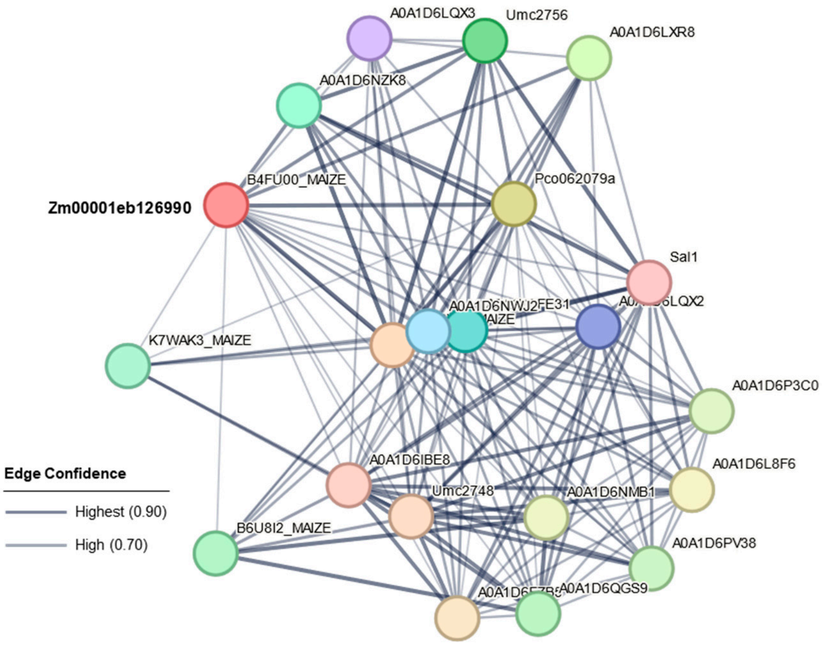

Figure S5. Prediction of endogenous interacting proteins of ZmSNF7 protein (B4FU00) in maize. All data were obtained from the STRING database.

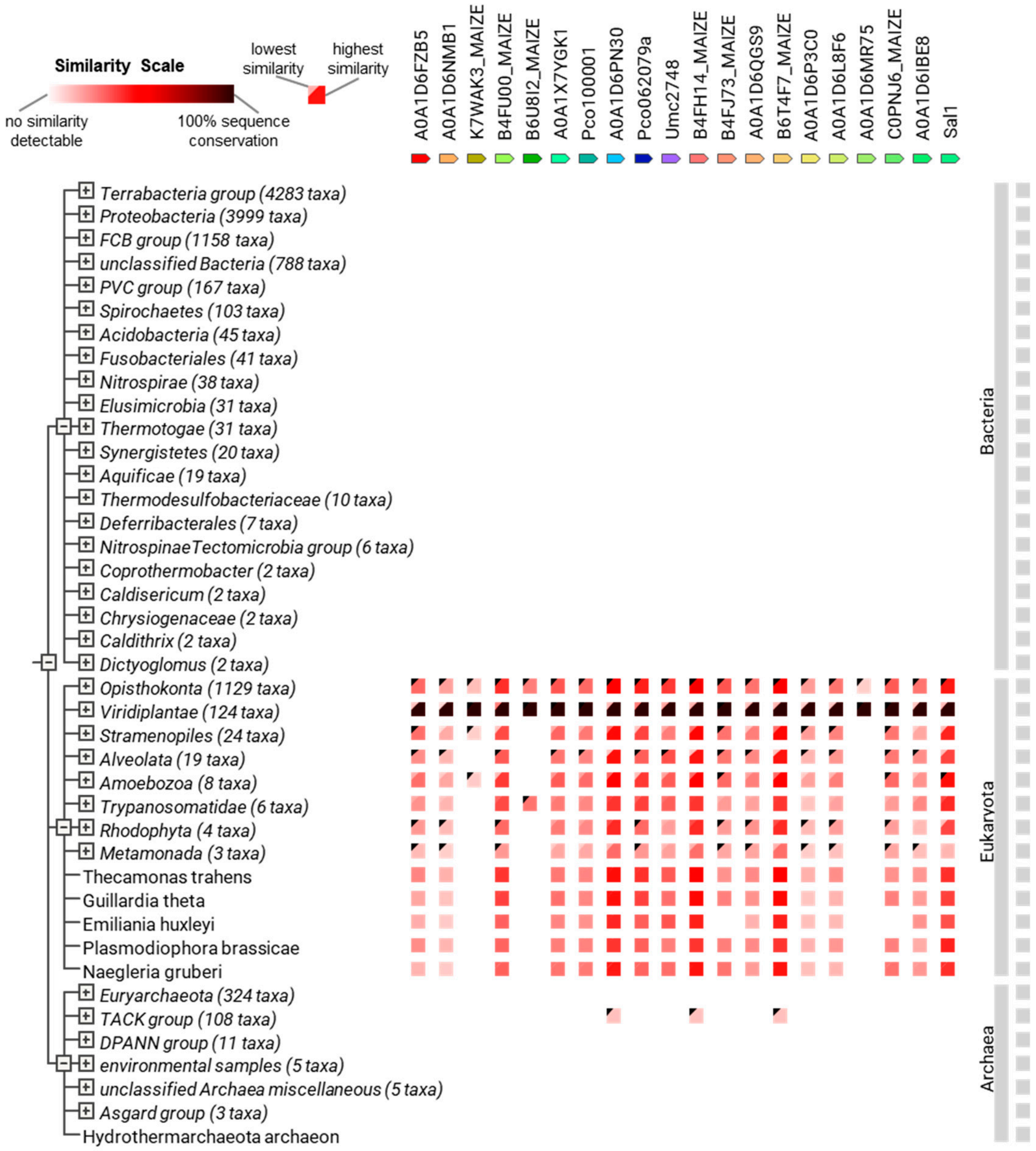

Figure S6. Heatmap of co-occurrence and evolutionary distribution of *ZmSNF7* family proteins. The color gradient indicates the level of sequence similarity (from no detectable homology in white to 100% sequence conservation in dark red/black). The data were generated using the STRING database.

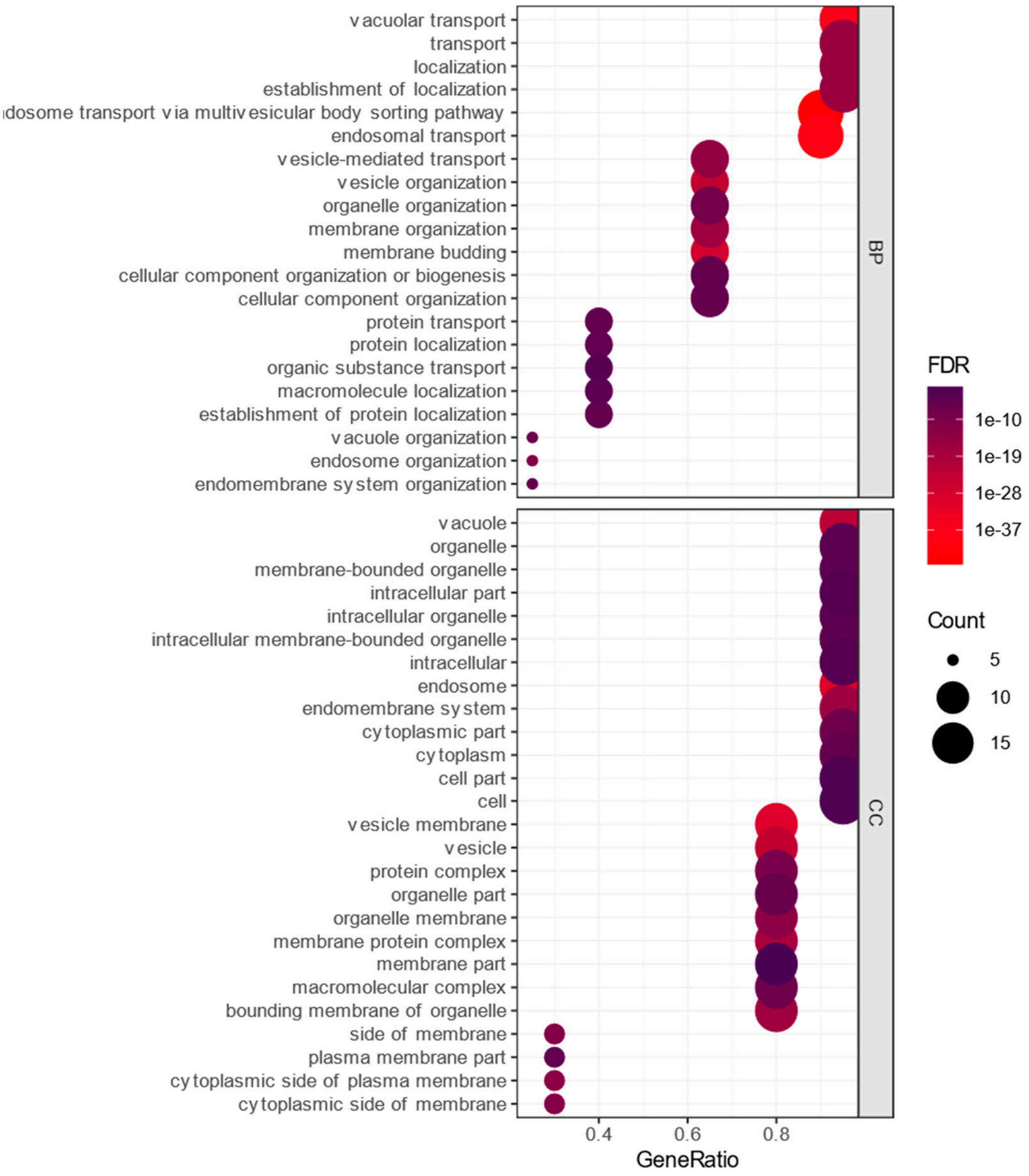

Figure S7. GO enrichment analysis of *ZmSNF7s*.

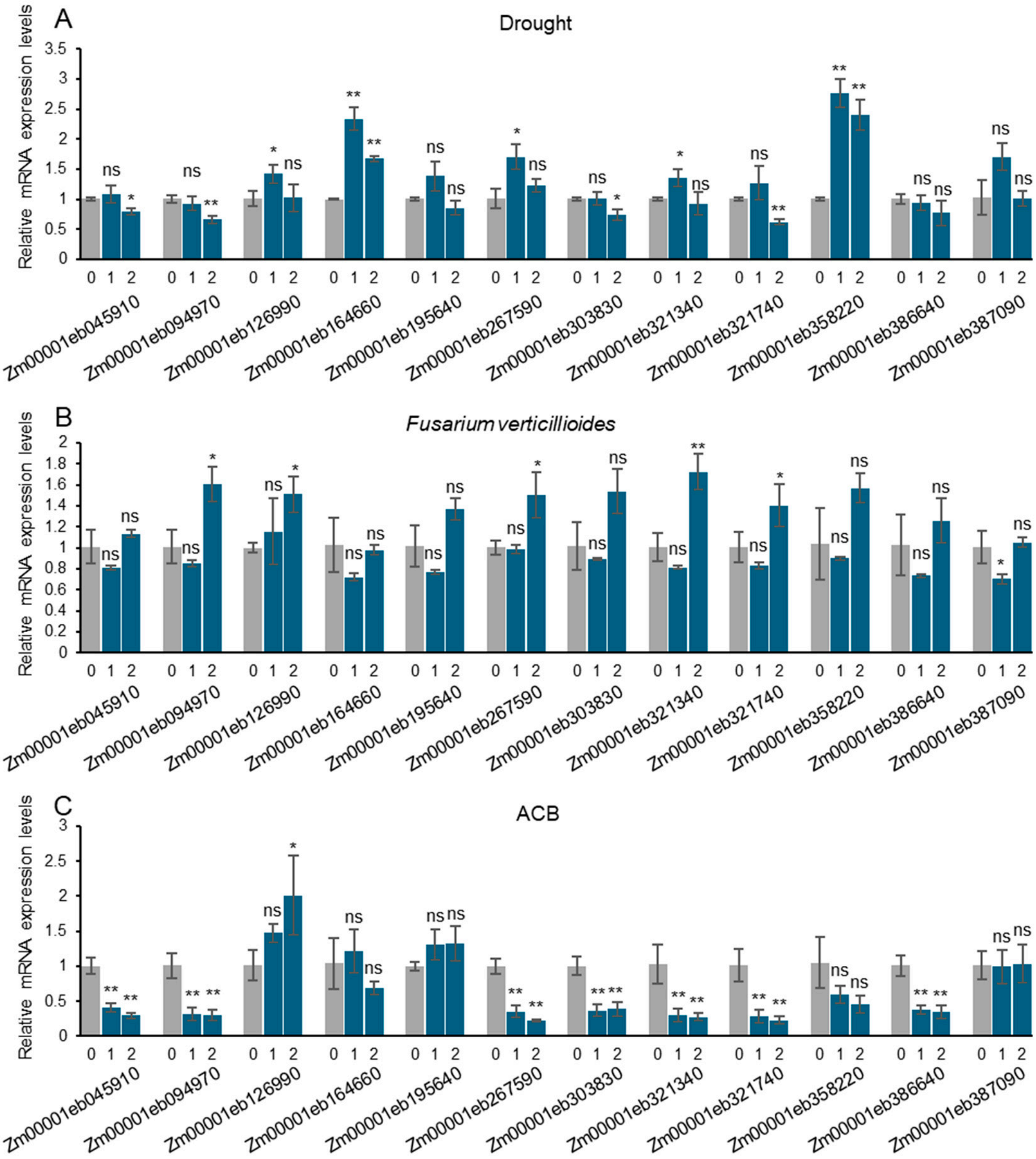

Figure S8. Relative expression levels of *ZmSNF7s* genes under three stress treatments. (A) The relative expression of *ZmSNF7s* genes under drought stress. 0 represents the control group, 1 and 2 indicate 1-day and 2-day stress treatments, respectively. (B) The relative expression of *ZmSNF7s* genes under *Fusarium verticillioides* stress. 0 represents the control group, 1 and 2 correspond to 1-day and 2-day treatments, respectively. (C) The relative expression of *ZmSNF7s* genes under Asian corn borer (ACB) infestation stress. 0 represents the control group, 1 and 2 indicate 1-hour and 2-hour treatments, respectively. The expression level of each gene at each stress time point was statistically analyzed with the control group using Student's t-test. Asterisks indicate significant differences: * $p< 0.05$, ** $p < 0.01$; ns represents no significant difference ($p > 0.05$). Error bars represent the standard error of three biological replicates, and each biological replicate consisted of mixed samples from five maize seedlings.

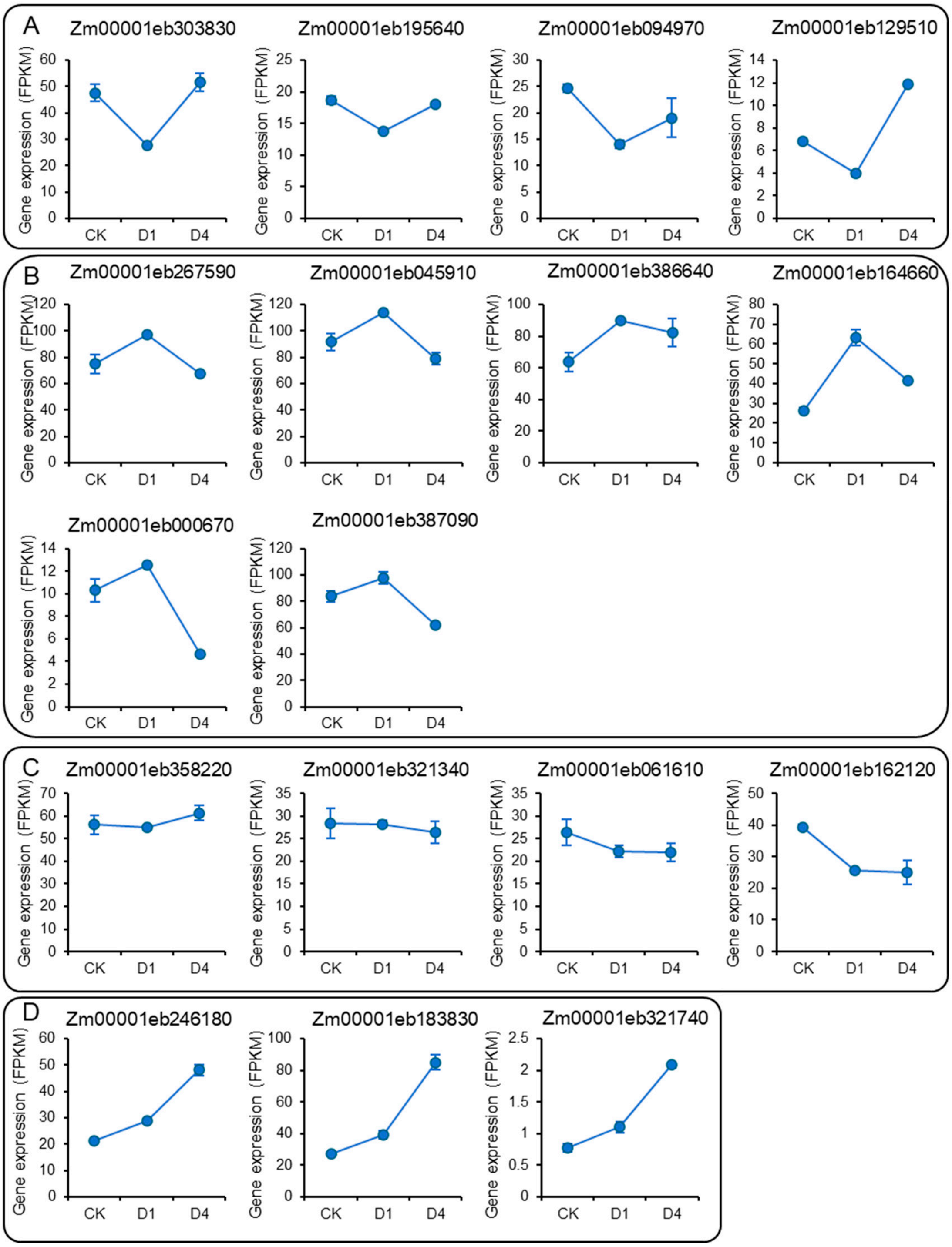

Figure S9. FPKM expression dynamics of *ZmSNF7* family genes in maize under drought stress.

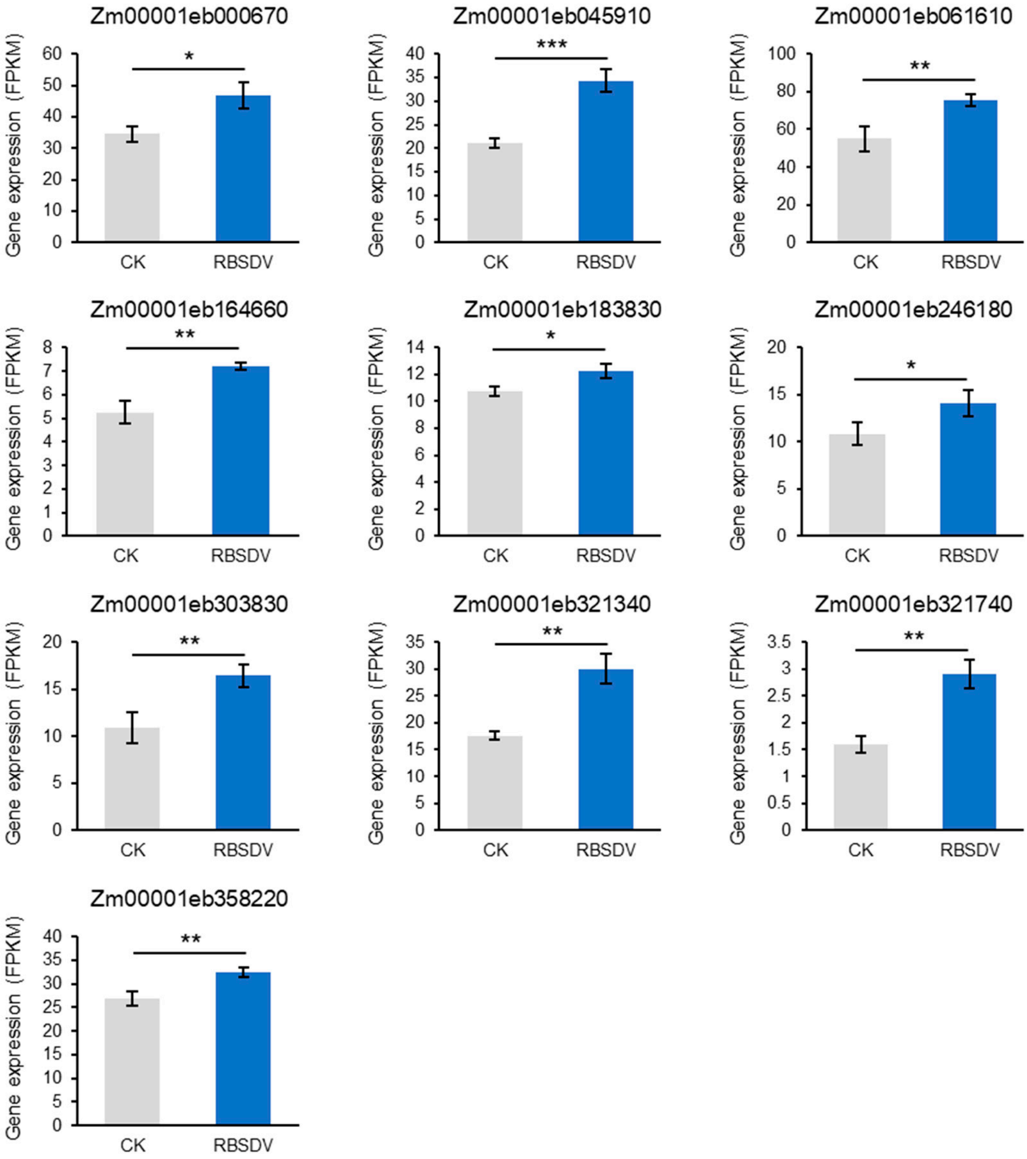

Figure S10. FPKM expression dynamics of *ZmSNF7* family genes in maize under RBSDV stress.

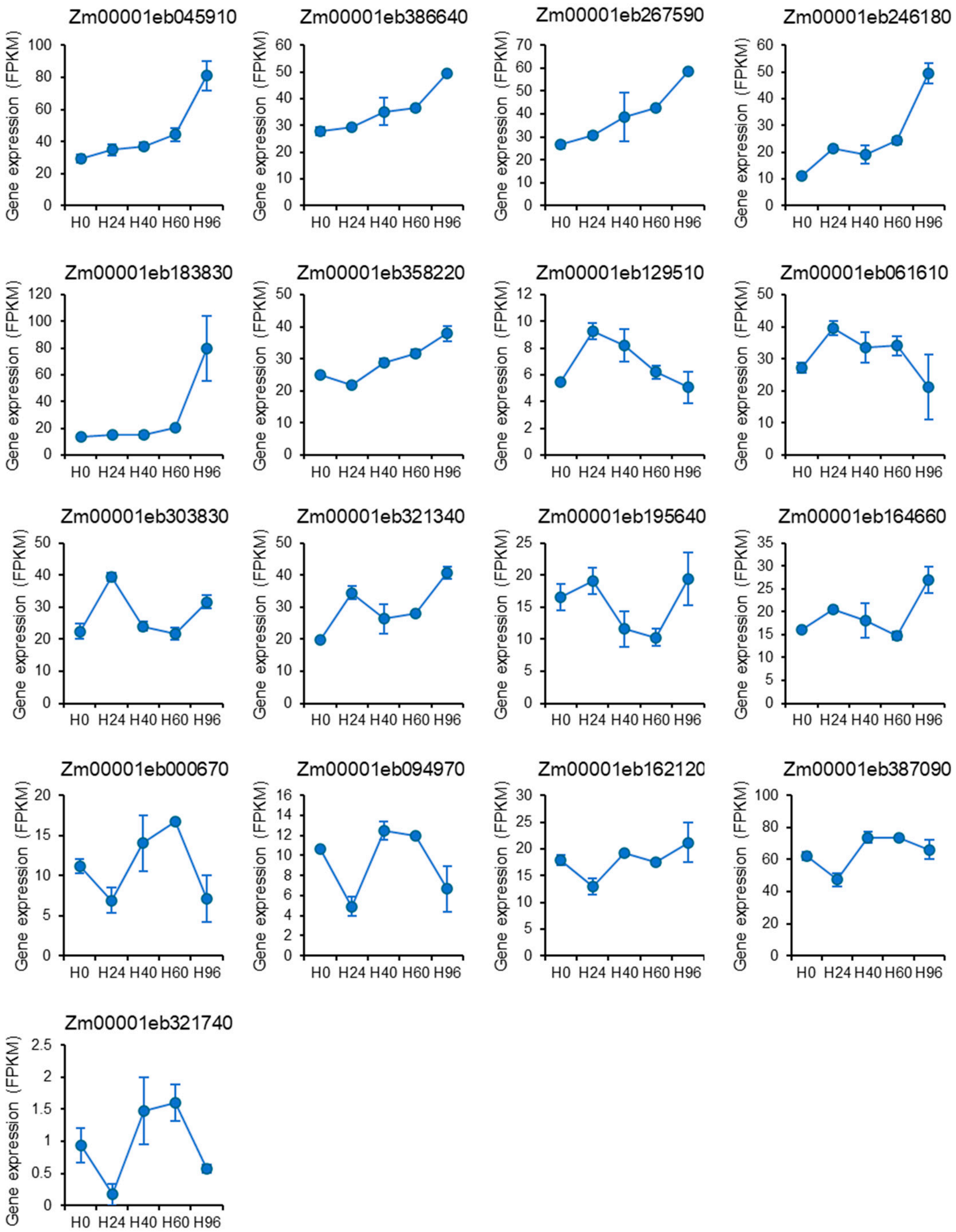

Figure S11. FPKM expression dynamics of *ZmSNF7* family genes in maize under *C. graminicola* stress.

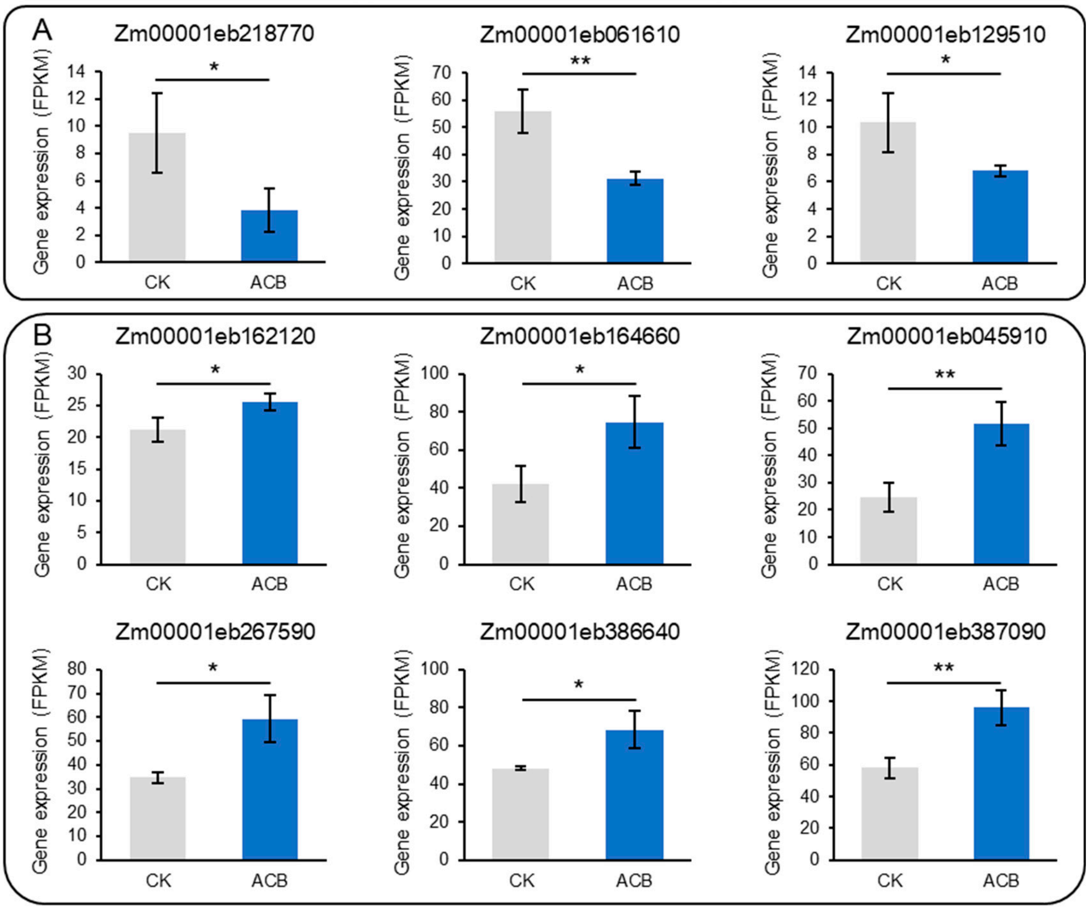

Figure S12. FPKM expression dynamics of *ZmSNF7* family genes in maize PHW03 under Asian corn borer (ACB) stress.
